# Structural plasticity enables evolution and innovation of RuBisCO assemblies

**DOI:** 10.1126/sciadv.adc9440

**Published:** 2022-08-26

**Authors:** Albert K. Liu, Jose H. Pereira, Alexander J. Kehl, Daniel J. Rosenberg, Douglas J. Orr, Simon K. S. Chu, Douglas M. Banda, Michal Hammel, Paul D. Adams, Justin B. Siegel, Patrick M. Shih

**Affiliations:** ^1^ Department of Plant and Microbial Biology, University of California, Berkeley, Berkeley, CA 94720, USA.; ^2^ Environmental Genomics and Systems Biology Division, Lawrence Berkeley National Laboratory, Berkeley, CA 94720, USA.; ^3^ Biochemistry, Molecular, Cellular and Developmental Biology Graduate Group, University of California, Davis, Davis, CA 95616, USA.; ^4^ Technology Division, Joint BioEnergy Institute, Emeryville, CA 94608, USA.; ^5^ Molecular Biophysics and Integrated Bioimaging Division, Lawrence Berkeley National Laboratory, Berkeley, CA, 94720, USA.; ^6^ Biophysics Graduate Group, University of California, Davis, Davis, CA, USA.; ^7^ Graduate Group in Biophysics, University of California, Berkeley, Berkeley, CA 94720, USA.; ^8^ Lancaster Environment Centre, Lancaster University, Lancaster LA1 4YQ, UK.; ^9^ Department of Bioengineering, University of California, Berkeley, Berkeley, CA 94720, USA.; ^10^ Genome Center, University of California, Davis, Davis, CA 95616, USA.; ^11^ Chemistry Department, University of California, Davis, Davis, CA 95616, USA.; ^12^ Department of Biochemistry and Molecular Medicine, University of California, Sacramento, Sacramento, CA 95616, USA.; ^13^ Feedstocks Division, Joint BioEnergy Institute, Emeryville, CA 94608, USA.; ^14^ Innovative Genomics Institute, University of California, Berkeley, Berkeley, CA 94720, USA.

## Abstract

Oligomerization is a core structural feature that defines the form and function of many proteins. Most proteins form molecular complexes; however, there remains a dearth of diversity-driven structural studies investigating the evolutionary trajectory of these assemblies. Ribulose-1,5-bisphosphate carboxylase-oxygenase (RuBisCO) is one such enzyme that adopts multiple assemblies, although the origins and distribution of its different oligomeric states remain cryptic. Here, we retrace the evolution of ancestral and extant form II RuBisCOs, revealing a complex and diverse history of oligomerization. We structurally characterize a newly discovered tetrameric RuBisCO, elucidating how solvent-exposed surfaces can readily adopt new interactions to interconvert or give rise to new oligomeric states. We further use these principles to engineer and demonstrate how changes in oligomerization can be mediated by relatively few mutations. Our findings yield insight into how structural plasticity may give rise to new oligomeric states.

## Teaser

Retracing the evolution of extant and ancestral RuBisCO reveals the origins and molecular basis of oligomerization.

## INTRODUCTION

The vast majority of proteins oligomerizes into higher-order molecular assemblies; however, the phenomenon of protein oligomerization has long remained paradoxical, despite its prevalence in nature. Two contrasting—although not mutually exclusive—modes for the evolution of oligomerization are commonly rationalized. In one, the assembly of a fixed number of subunits is required for protein function (e.g., substrate binding and catalysis), with selection driving the adoption of oligomeric states over time to maintain activity (*1*, *2*). In the other, mutational trends result in a propensity to oligomerize, albeit decoupled from catalytic activity (*3*–*5*). Given that alterations to protein structure enable and/or potentiate new functions, understanding how new oligomeric states originate is a fundamental aspect of protein evolution. Although there has been great interest in elucidating the molecular factors driving new forms of oligomerization, these studies require the comprehensive characterization of entire protein families across time and phylogeny; however, most structural studies have focused on small subsets to single representatives of protein families (*5*). Without first-order knowledge describing the distribution and diversity of protein oligomerization, we have been largely unable to discern the degree of oligomeric drift that occurs during the evolutionary process and how it may contribute to new functional commitments of proteins.

Our current understanding of the prevalence and extent of quaternary structure plasticity has primarily been determined through large-scale analyses of existing structural databases (*6*–*8*). Global investigation of these databases has yielded important insights into interface identities, prediction of quaternary structure, and evolutionary pathways taken (*6*–*8*). However, the inherently slow and laborious nature of structural determination hampers our ability to more rigorously and systematically assess trends in oligomerization; selective sampling of proteins amenable to purification and crystallization limits the throughput of solved structures, and crystallographic artifacts can result in misassignment of quaternary structure altogether (*6*, *9*). Furthermore, the use of existing structures precludes the investigation of evolutionary intermediates, as this would require the coupling of ancestral sequence reconstruction with structural determination to sample across time and phylogeny (*5*). Broader trends can be assessed through analyses of deposited protein structures; however, this will always entail biased samplings that may be too sparse for understanding transitions in quaternary structure at a finer scale of evolution (*5*). Thus, there exists a need to identify model protein families amenable to biochemical investigations to yield key insights into the plasticity and trajectories of protein oligomeric state.

Ribulose-1,5-bisphosphate carboxylase-oxygenase (RuBisCO) is not only one such enzyme where biological function is predicated upon oligomeric state but is also capable of adopting multiple assemblies. All RuBisCOs are composed of a core dimeric scaffold with two monomers arranged in *C*_2_ symmetry, which is requisite for forming the active site and enabling catalytic activity; however, complexes from dimeric building blocks can assemble into higher-order structures. The vast majority of research has centered on form I RuBisCOs, as the biological source of nearly all organic carbon on Earth, yet the evolutionary events leading to its unique hexadecameric assembly—eight large and eight small subunits—remain elusive (*10*–*12*). In contrast, all other forms of RuBisCO across the tree of life lack small subunits and instead assemble as a variety of homomeric complexes. In particular, representatives of form II RuBisCOs have been shown to assemble as either dimers or hexamers, in which the hexamers are composed of base dimers arranged in *D*_3_ symmetry. Thus, this offers a unique system in which to study the evolution and transitions of oligomerization of a related enzyme lacking the strict structural requirements of the form I enzyme (*13*–*16*). Here, we investigate the diversity and evolutionary trajectory of oligomerization in form II RuBisCOs, revealing a trend of structural plasticity that underlies the interconversion between, and innovation of, multiple oligomeric states.

## RESULTS

### Diversity-driven sampling across extant RuBisCO reveals complex history of oligomeric state

**Fig. 1.**
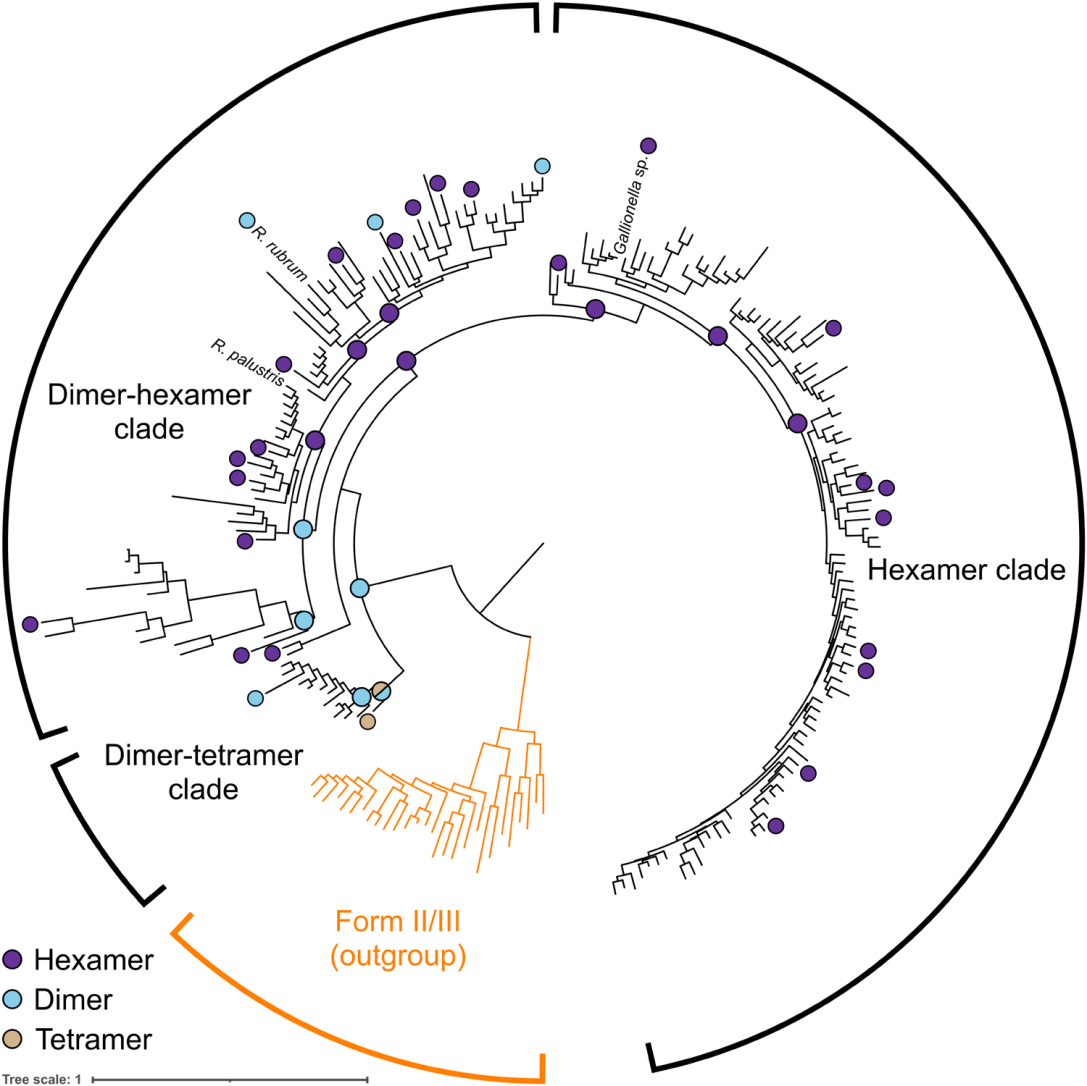
Diversity-driven sampling reveals plasticity of RuBisCO oligomeric state. Phylogenetic tree of form II RuBisCO, form II/III serving as outgroup. Selection of presented sequences detailed in Materials and Methods. Oligomeric states of characterized extant enzymes are indicated at tips, and those of ancestral enzymes are indicated at corresponding nodes.

To better understand the phylogenetic distribution of oligomeric states found within form II RuBisCO, we structurally characterized 28 candidates spread across the phylogeny (Fig. 1, fig. S1, and table S1). From a recent library of form II RuBisCOs, all homologs were heterologously expressed, purified, and analyzed by size exclusion chromatography coupled with small-angle x-ray scattering and multiangle light scattering (SEC-SAXS-MALS) (*16*–*19*). While rudimentary oligomeric state determination can be conducted by SEC alone (*16*), the application of SEC-SAXS-MALS for this purpose permits structural differentiation via comparisons of x-ray scattering profiles of different assemblies, as well as additional support from measured molecular weights (*17*–*19*). From the collected SAXS profiles and estimated molecular weights, we observed both dimers and hexamers, with 23 of 28 adopting the hexameric state (Fig. 1, fig. S2, and table S1). Notably, we collected SAXS and MALS data from a tetrameric enzyme, representing an entirely new oligomeric state of RuBisCO that has never been structurally characterized, supporting a high level of quaternary diversity within form II RuBisCOs (fig. S2 and table S1) (*16*). It is commonly believed that form II RuBisCO exist primarily as dimers; this is largely assumed because the first solved crystal structure of a RuBisCO was a dimeric form II RuBisCO from *Rhodospirillum rubrum* (*13*). However, more recently, the crystal structure of two hexameric structures has also been described (*14*, *15*). By taking a phylogenetic approach to characterizing this entire protein family, we demonstrate that the vast majority is actually hexameric (Fig. 1). Our findings illustrate the need for diversity-driven studies to correct preconceived biases resulting from sparse structural sampling in our understanding of how protein structures and entire protein families evolve over time.

Upon mapping the characterized oligomeric states onto the phylogeny, we uncovered three distinct patterns of oligomerization representing parallel evolutionary trajectories. One clade, here referred to as the hexamer clade, is entirely composed of hexamers, including a previously characterized Gallionellaceae enzyme (Fig. 1) (*15*). In contrast, the dimer-hexamer clade displays several dimeric enzymes interspersed between hexamers, highlighting the structural plasticity of form II RuBisCOs in this clade (Fig. 1). These structural reversions provide a unique case study to demonstrate how the dimer-hexamer clade is not structurally entrenched and thus has the ability to drift from one state to another. Notably, this clade includes the benchmark form II RuBisCO from *R. rubrum*, as well as another structurally characterized hexamer from *Rhodopseudomonas palustris* (*13*, *14*). Last, the dimer-tetramer clade is composed of dimers and the tetrameric RuBisCO, providing a glimpse into how nature has been able to evolve and innovate new oligomeric states (Fig. 1). Overall, our diversity-driven structural characterization across form II RuBisCOs reveals three different clades with three unique evolutionary histories: (i) structural entrenchment, (ii) reversible transition states, or (iii) innovation of entirely new oligomeric states.

### Reconstructing evolutionary trajectories across time elucidates plasticity of oligomeric state

To expand beyond sampling extant sequences, we recapitulated the evolutionary histories of these three different clades by characterizing the ancestral nodes across the form II phylogeny. We synthesized and characterized 12 ancestral sequence reconstruction enzymes in a manner similar to the extant form II RuBisCOs. The most recent common ancestor (MRCA) of all form II RuBisCOs (node 8) was dimeric, reinforcing the most parsimonious scenario of a dimeric origin of form II RuBisCO (Fig. 1 and fig. S3). Notably, the dimer-tetramer clade MRCA (node 9) adopts both a dimeric and tetrameric state in solution as captured by SEC-SAXS-MALS (Fig. 1 and fig. S3). The subsequent sister node 10 forms a dimer, representing the origin of the dimers within the dimer-tetramer clade. The biphasic assemblies of node 9 demonstrate the structural plasticity of form II RuBisCOs, as it reprints an evolutionary intermediate that has the propensity to form either a dimer or tetramer before the eventual commitment to either trajectory. This evolutionary plasticity is not observable from solely sampling extant enzymes, highlighting the need for ancestral enzyme characterization to visualize oligomeric interconversion within structurally plastic enzyme families.

In conjunction with the oligomeric state of the form II MRCA, analysis of nodes within the dimer-hexamer clade revealed multiple independent interconversion events. From the most ancestral dimer, an intermediary hexamer (node 23) underwent a reversion event resulting in the ancestral dimer preceding the dimer-hexamer clade (node 127) (Fig. 1 and fig. S3). From node 127, the dimer then formed and maintained the hexameric state over several branch points, before reverting once more into extant dimers (Fig. 1 and fig. S3). This clade reinforces the idea that the oligomeric state in some protein families may be quite plastic, allowing for reversions and transitions between different states. This is best demonstrated by a pair of two closely related homologs from *Insolitispirillum peregrinum* and Rhodospirillaceae bacterium BRH_c57 (76.3% amino acid identity), which form a dimer and hexamer, respectively. This is in contrast with the hexamer clade, whose ancestral enzymes at nodes 24, 27, and 28 were indeed hexameric as well (Fig. 1 and fig. S3). The hexamer clade suggests that there is some biochemical purpose that has entrenched this clade as hexamers, whereas the dimer-hexamer clade is free of those quaternary structure restrictions.

These observations provide insight as to how evolutionary trajectories may affect patterns of oligomerization of phylogenetically related enzymes: Entire clades can adhere to a singular oligomeric state, or plasticity can enable free interconversion over time. Although it has been suggested that a ratchet-like evolution of oligomeric state may drive proteins into higher-order assemblies mediated by hydrophobic interactions, not all homomeric or heteromeric complexes form via solely hydrophobic patches (*3*). RuBisCO offers an interesting counterexample where homomeric complexes form via solvent-accessible polar interactions, which underpins the flexibility of oligomeric state in the dimer-hexamer clade. With no known functional constraint between dimers or hexamers, the dimer-hexamer clade appears to have the oligomeric plasticity to explore and interconvert between both states, whereas the hexamer clade has been captured in a sole oligomeric state, likely stemming from an uncharacterized functional pressure. The extent and pervasiveness of proteins that are amenable to this level of quaternary structure freedom may be hard to determine. However, our analyses provide an important case study on how structural plasticity may enable protein drift through both sequence space and oligomeric state while innovating new forms and functions. This could explain the two states observed in the dimer-hexamer clade and the tetramer in the dimer-tetramer clade. However, a functional role may still result in oligomeric entrenchment, resulting in the widespread adoption of a singular oligomeric state (e.g., hexamer clade).

### Evolutionary innovation of a tetrameric RuBisCO that co-opts a unique dimer-dimer interface

Previously, RuBisCO has only been described to form dimers and assemblies composed of repeating dimers, which arrange into dihedral ring-like structures around a central solvent channel (e.g., hexamers, octamers, and decamers), with previous work suggesting the existence of a tetrameric assembly from the organism *Sulfurivirga caldicuralii* (*16*). SEC-SAXS-MALS analysis on the *S. caldicuralii* enzyme revealed a molecular weight of 218.3 kDa, in agreement with a proposed composition of four large subunits of approximately 50 kDa each (table S1). The collected SAXS curve did not match trends observed from either dimeric or hexameric RuBisCOs, further suggesting that the tetrameric state is distinct from other form II structures (fig. S2). In addition, the SAXS curve did not fit a tetrameric structure generated by removing two dimers from the octameric core of a form I RuBisCO, thus informing us that the assembly of the *S. caldicuralii* tetramer is distinct from that found within the octameric RuBisCO (fig. S4, A and B).

**Fig. 2.**
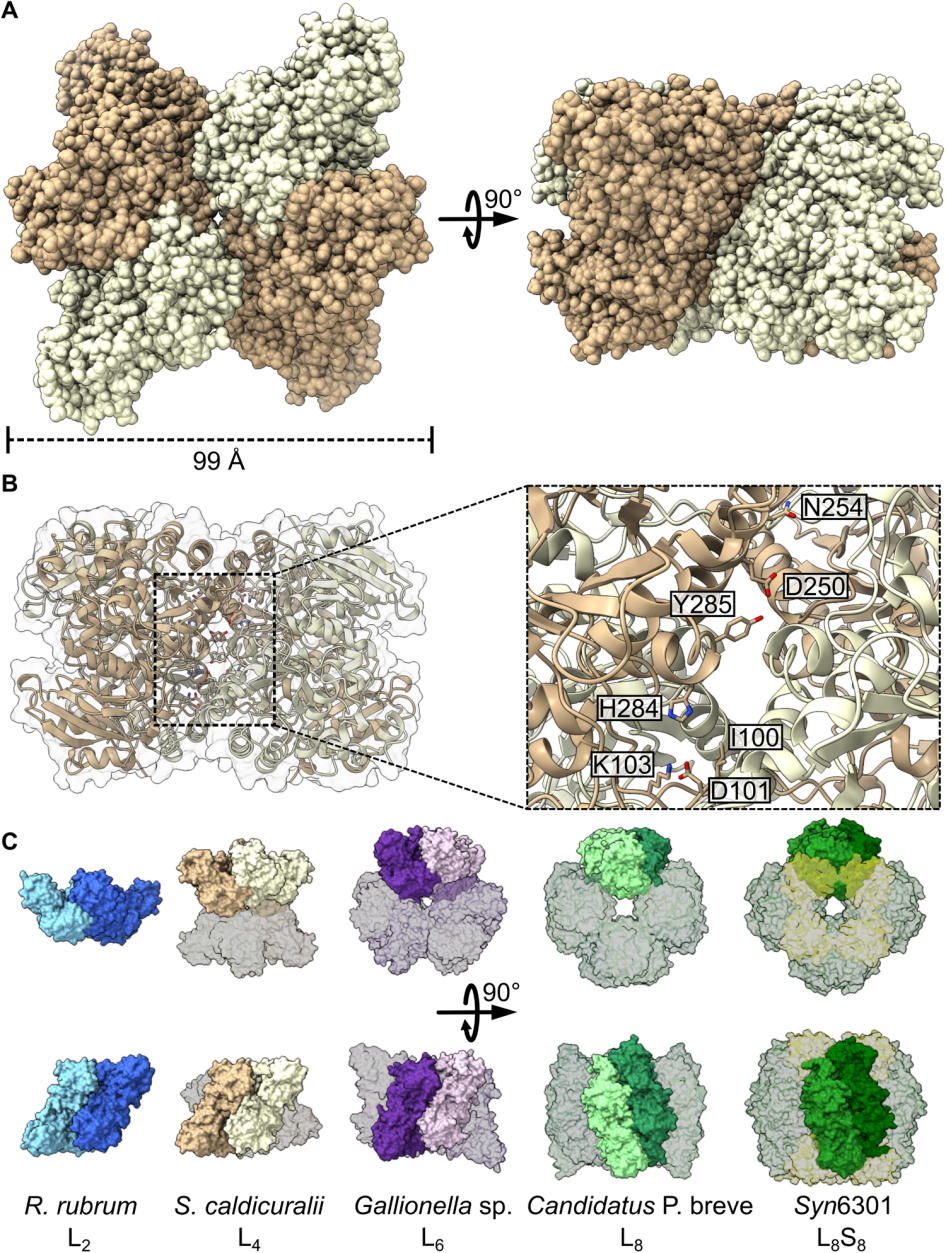
Crystal structure of a tetrameric RuBisCO. (**A**) Structure of *S. caldicuralii* RuBisCO resolved at 1.7 Å. (**B**) Interface cutaway of *S. caldicuralii* tetramer with candidate residues indicated. (**C**) Comparison of RuBisCO oligomeric states illustrating dimer positioning within a multimer. Form II dimer, tetramer, and hexamer are shown alongside form I′ octamer and form I hexadecamer. Protein Data Bank (PDB) codes (left to right): 5RUB, 7T1C, 5C2C, 6URA, and 1RBL.

To better understand the oligomeric state of *S. caldicuralii* RuBisCO, we solved its crystal structure at 1.7-Å resolution, clearly displaying its tetrameric assembly (Fig. 2A). The arrangement of the pair of dimers precludes the formation of the aforementioned central solvent channel (fig. S4C). Identification of interface residues revealed a compacted interface aligned more closely to the center of each dimer and distinct from that of the hexamer’s (Fig. 2B and fig. S5). This illustrates the means by which new oligomeric states can be innovated over the course of structural drift, as the tetramer is differentiated both phylogenetically and structurally from the hexamer, thus precluding the use of the larger oligomeric state as the template. In conjunction with our phylogenetic analyses, this observation highlights the unique assembly of the tetramer, as its early divergence from the remainder of form II RuBisCO precedes the innovation of the hexameric state yet remains maintained after the divergence from ancestral node 9 into the remainder of the dimer-tetramer clade.

Moreover, when compared to the octameric cores of form I and I′ assemblies, it becomes apparent that the combination of two tetramers would not yield a conventional octamer (Fig. 2C). Numerically, an octameric protein (a tetramer of functional dimers) could be assembled from two tetramers (dimers of dimers), in accordance with our understanding of oligomeric assembly (*7*, *20*). However, RuBisCO dimers within an octameric core are vertically aligned in parallel, whereas the *S. caldicuralii* RuBisCO’s central axis results in the observed angled assembly, thus precluding the formation of form I–like octamers from form II tetrameric RuBisCOs. This further illustrates the differences between the evolutionary trajectory of form I and form II oligomeric state, as the geometric differences between a pair of form I dimers and the form II tetramer suggest the independent innovation of the tetrameric state.

Structural plasticity has been proposed to affect the oligomeric state of enzyme families in two distinct ways: (i) Large geometric changes can be buffered by plasticity and result in the maintenance of oligomeric state, or (ii) plasticity can underpin geometric flexibility and give rise to multiple oligomeric states (*21*). Form I RuBisCO may represent an example of the former situation, as it remains highly constrained by its base octameric assembly, thus resulting in minor changes to the angle of dimers within the octamer without changes in the entirety of its oligomeric state. In contrast, we demonstrate that form II RuBisCO falls into the latter category, where a highly plastic ancestral dimer may have fortuitously bound a second dimer and gave rise to tetramerization, while subsequent evolution of singular dimers produced the precursor interfaces necessary for hexamerization. Ultimately, the tetrameric form of RuBisCO exemplifies how the structural plasticity of proteins enables the innovation of entirely new oligomeric states through the recruitment of surface residues to mediate protein-protein interactions.

### Structural plasticity enables reversions to simpler oligomeric states

**Fig. 3.**
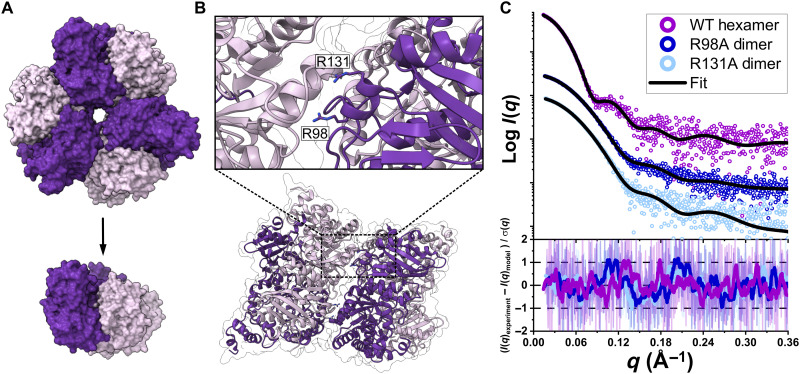
Hexamers can readily form dimers through mutations of residues coordinating the interdimer interface. (**A**) Modeling disruptions at the interdimer interface of the hexameric *Gallionella* sp. structure (PDB: 5C2C) to shift its oligomeric state from hexamer to dimer. (**B**) Interface cutaway indicating candidate residues. (**C**) SAXS curves of experimental data for wild-type (WT) enzyme, R98A, and R131A mutants and theoretical fit models for hexameric and dimeric states (PDB: 5C2C and 5RUB, respectively). Fit residuals shown below.

To investigate the hypothesis that molecular complexes are subject to ratchet-like evolution that entrenches oligomeric states of proteins (*3*), we tested how easily form II proteins could revert from higher-order hexamers to the simpler dimeric state. To identify the specific interface residues involved in higher-order assembly of RuBisCO, we used Protein Contacts Atlas to analyze the interdimer interface of a previously characterized hexameric Gallionellaceae enzyme (GWS1B) (Fig. 3A and fig. S6, A and B) (*15*, *22*). From a list of computed atomic interactions, we found two arginine residues at positions 98 and 131 capable of forming multiple interactions across the interface, including a potential salt bridge with an aspartic acid residue at position 256 (Fig. 3B and fig. S6B). Using the *Gallionella* sp. enzyme as a template, we conducted sequence conservation analysis to further analyze the composition and maintenance of the hexameric RuBisCO interface (fig. S7A). Across all extant hexamers identified from our characterization experiments, the R98 residue proved to be more conserved than R131, although neither proved to be especially variable in comparison to a less conserved residue, such as Y358 (fig. S7B). However, when comparing patterns within clades, the residue identity of position 131 is highly variable in the dimer-hexamer clade compared to the hexamer clade, wherein both R98 and R131 are highly conserved (fig. S7, C and D). The variability in interface residue conservation across clades demonstrates the mechanisms of differentiation between the dimer-hexamer and hexamer clades, as R131 may serve as one such residue that strengthens the hexameric state within the hexamer clade, whereas the plasticity within the dimer-hexamer clade resulted in more variable identities at that same position.

To query the contribution of R98 and R131 to the maintenance and stability of the interdimer interface, we conducted alanine substitutions at both positions and characterized the point mutant enzymes in the same manner as its wild-type counterpart. Notably, both the R98A and R131A mutants adopted the dimeric state, as verified by SEC-SAXS-MALS (Fig. 3C). Analysis by protein thermal shift assays revealed a decrease in thermal stability for both mutant dimers relative to the wild-type, with R98A and R131A fully denaturing at 10.5° and 12.5°C, respectively, lower than the wild type (fig. S8). These findings are contrary to conventional perspectives on the strength and maintenance of oligomeric state, as a single-residue substitution resulted in loss of a higher-order assembly, although it remained structurally viable in its base state as opposed to an anticipated critical destabilization of the entire enzyme (*3*, *23*, *24*). Mutational ratchet-based oligomerization is considered irreversible because of the nature of its mechanism, as a disadvantageous property is thought to be conferred to composite subunits were they to be isolated from one another. However, we demonstrate that exposure of the buried hexameric interdimer interface does not result in catastrophic destabilization of the enzyme, suggesting that the irreversibility of higher-order oligomerization may be overruled by highly plastic evolutionary trajectories that enable interconversion events akin to our experiments.

### Oligomerization tunes RuBisCO activity and kinetic parameters

Although the residues defining RuBisCO dimer-dimer assembly are distal to the active site, we hypothesized that minor perturbations to the core dimer that still result in marked changes in quaternary state may affect the kinetic parameters of the enzyme. It has been previously demonstrated that these distal mutations can affect the enzymatic properties of a wide variety of enzymes (*25*, *26*); thus, we investigated the specific implications of oligomeric disruption on RuBisCO catalysis. We measured the kinetic parameters of the two mutant R98A and R131A enzymes (Table 1). Because of RuBisCO’s dual carboxylase and oxygenase activities, measured parameters include turnover numbers (*k*_cat_^C^ and *k*_cat_^O^, respectively), Michaelis constants for CO_2_ and O_2_ (*K*_C_ and *K*_O_), and the RuBisCO specificity factor (*S*_C/O_). Both mutants displayed decreased *k*_cat_^C^, by approximately 30% for R98A and approximately 22% for R131A relative to the wild type (Table 1). However, the mutant enzymes displayed an increase in specificity factor, with *S*_C/O_ values approximately 1.17 times higher in R98A and 1.13 times higher in R131A than the wild type (Table 1). In light of the modest changes to *K*_C_ and *k*_cat_^O^, the change in specificity appears to be largely driven by a markedly decreased affinity for oxygen as a substrate, with *K*_O_ values being approximately 1.68 times higher in R98A and 2.15 times higher in R131A than the wild type (Table 1).

**Table 1.** Dimers formed from hexamers demonstrate how distal mutations from the active site mediate enzymatic tradeoffs and fine tune kinetic properties of RuBisCO. Values are means ± SEM with *n* indicated in brackets.

**RuBisCO**	**Oligomeric state**	***k*_cat_^C^ (s^−1^)**	***K*_*C*_ (μM)**	***S*_C/O_**	***k*_cat_^O^ (s^−1^)**	***K*_O_ (μM)**
*Gallionella* sp. wild-type	L_6_	15.7 ± 0.9 (5)	172 ± 29 (5)	22.0 ± 1.3 (5)	0.38	92 ± 15 (4)
R98A	L_2_	11.1 ± 1.2 (4)	170 ± 25 (4)	25.7 ± 1.8 (6)	0.39	155 ± 16 (4)
R131A	L_2_	12.3 ± 0.9 (5)	198 ± 12 (4)	24.9 ± 0.9 (6)	0.50	198 ± 21 (4)

While the RuBisCO interdimer interface is distinct from the active site, the effects of mutational disruption of oligomeric state on catalytic activity were previously unknown. These experiments demonstrate that catalytic activity is maintained in the absence of the wild-type quaternary structure. In comparison to existing kinetic measurements for other form II RuBisCO, note that despite exhibiting decreased *k*_cat_^C^ values of the R98A and R131A mutants, both are still extremely high values, ranking within the top seven fastest RuBisCO ever studied: The fastest *Gallionella* sp. enzyme has a measured *k*_cat_^C^ of 22.2 s^−1^, bookended by the *R. rubrum* enzyme with a measured *k*_cat_^C^ of 6.6 s^−1^ (*16*). In addition, the wild-type GWS1B *Gallionella* sp. enzyme is the third fastest form II enzyme ever measured, surpassing the *Hydrogenovibrio marinus* dimer at 15.6 s^−1^ (*16*). In light of these considerations, the observation that complete reversion of the hexameric state to the dimeric state resulted in relatively minimal changes to most kinetic parameters is of great interest, as this suggests that the innovation of oligomeric states within the form II evolutionary trajectory may have incurred minimal functional penalty.

### Engineering increased oligomeric complexity

**Fig. 4.**
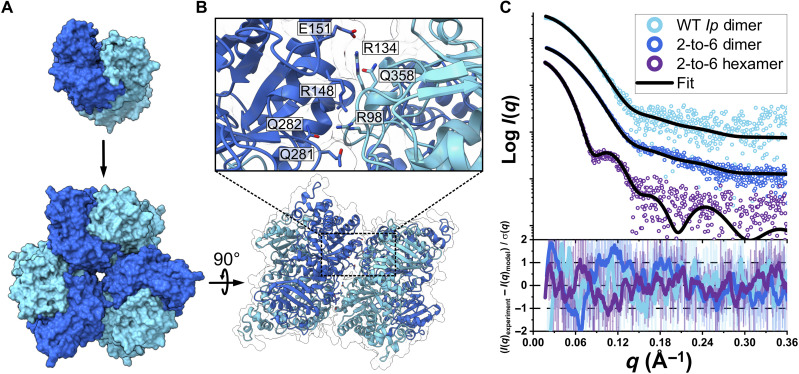
Structurally guided engineering recapitulates dimer-to-hexamer oligomeric transition. (**A**) Modeling of the interdimer interface to convert the dimeric *I. peregrinum* RuBisCO into a hexamer. (**B**) Interface cutaway of introduced mutations in the hexameric *I. peregrinum* homology model to engineer a network of side chain interactions to mediate an oligomeric shift to hexamerization. (**C**) SAXS curves of experimental data for wild-type and engineered *I. peregrinum* enzyme and theoretical fit models for both hexameric and dimeric states present in the same characterized sample [PDB: 7T1J and homology model of *I. peregrinum* (*Ip*) dimer, respectively]. Fit residuals are shown.

To further query the structural plasticity of form II RuBisCO, we tested how readily we could introduce surface mutations to the enzyme to engineer higher-order assemblies of RuBisCO from the base dimer. We developed a Rosetta-based computational pipeline to model the transition of a dimer to a hexamer, dubbed “2-to-6.” Two closely related RuBisCOs from the dimer-hexamer clade were used as a template hexamer and a candidate dimer, where the dimer (*I. peregrinum*) and the hexamer (BRH_c57) share 76.3% sequence identity (fig. S9A). In addition, we solved the crystal structure of the BRH_c57 hexamer to identify the residues participating in its interdimer interface, in conjunction with Rosetta modeling of a mutant 2-to-6 *I. peregrinum* hexamer (Fig. 4A; fig. S9, A and B). Initially, simple mutational experiments were performed using the interface interactions derived from the BRH_c57 structure, although these did not result in an increase in oligomeric state. Thus, we used a more rigorous modeling and scoring protocol within Rosetta to screen 128 combinations of different interface residue mutants, with a total of seven residue substitutions (K98R, A134R, T148R, G151E, G281Q, T282Q, and G358Q) introduced into the *I. peregrinum* sequence based on the top candidate (Fig. 4B). The candidate 2-to-6 sequence was then expressed, purified, and characterized by SEC-SAXS-MALS, confirming the generation of a hexameric *I. peregrinum* RuBisCO. Of the seven substitutions, the R98, R148, and Q282 residue identities were also present in the hexameric sequence conservation analysis conducted previously, while the remainder was unique to the BRH_c57 enzyme. Notably, the G358Q mutation was predicted to position R134 and enable an interaction with E151—an interaction not observed in the original BRH_c57 interface (fig. S9C). Our engineered protein demonstrates how higher oligomeric states can be assembled through point mutations at the interdimeric interface, with further structural differentiation innovated by residue positioning.

Analysis of the SEC-SAXS-MALS sample revealed an unexpectedly bimodal SEC curve for the engineered 2-to-6 enzyme, suggesting a heterogenous population. A hexameric assembly was captured and verified by comparison of its SAXS scattering data with the BRH_c57 hexamer, but a second dimeric state was also present in the purified sample (Fig. 4C). The presence of both oligomeric states is akin to the behavior exhibited by the dimeric/tetrameric ancestral node 9, suggesting the capture of an intermediary transitional state before commitment to either the dimeric or hexameric state. While the 2-to-6 sequence ranked highest from Rosetta modeling, the distribution of dimeric and hexameric species in the experimental sample (approximately 25% hexamers and 75% dimers) suggests that residues not involved in hydrogen bonding at the interdimer interface may play a key role in enabling the complete transition to a hexameric state, in agreement with previous observations regarding the role of distant mutations in oligomerization (fig. S10) (*21*).

Our results demonstrate how a small number of residues—only seven mutations—can enable an increase in oligomeric state, providing insight into the requisite degree of plasticity necessary for innovation of larger oligomeric states. Recent studies have demonstrated how few mutations can be introduced to proteins, resulting in radical increases in oligomerization that are more accurately described as non-native protein fibrils (*27*–*29*). In comparison, our study focuses on understanding the structural basis underlying transitions between oligomeric states found in nature, as our mutational engineering of the 2-to-6 enzyme recapitulates the evolutionary trajectory taken by form II RuBisCOs when assembling hexamers from dimers. Although previous work has relied on the introduction of hydrophobic patches to enable the self-assembly of large protein complexes (*30*), we demonstrate that subtle structural differences of polar amino acids on a solvent-exposed surface can be used to dictate the proper formation of predicted interactions constituting protein-protein interfaces.

## DISCUSSION

Our understanding of the diversity, origins, and trajectories of protein oligomeric states has been largely incomplete because of the lack of diversity-driven studies required to properly assess how quaternary structure evolves over time. Our findings reveal an unprecedented level of structural plasticity underlying an assortment of unique evolutionary trajectories within a single protein family, ranging from structural entrenchment, interconversions, and innovation of new oligomeric states. The characterization of a previously unknown tetrameric form of RuBisCO best highlights how evolution continually explores sequence space and co-opts surface residues in the formation of entirely new oligomeric states. Because the majority of proteins form molecular complexes, the underappreciation of this higher form of structural plasticity may have larger implications on many other protein families, where quaternary structure can play a key role in drug targets (*31*, *32*), human diseases (*33*, *34*), and general function (*35*–*37*).

While mining protein structure databases has greatly advanced our understanding of the prevalence and trajectory of quaternary structure, there remains a lack of extant and ancestral coverage of evolutionary intermediates and the potential for structural plasticity therein (*6*, *7*, *9*). It has been previously hypothesized that sampling ancestral representatives from a protein family containing multiple oligomeric states may reveal occupation of different states at different points in time (*5*). Form II RuBisCOs offer an ideal model clade to demonstrate how systematic sampling of extant and ancestral enzymes allows a retracing of evolutionary trajectories between disparate oligomeric states. Notably, sampling and phylogenetic resolution across a single protein family were necessary to reveal interconversion and innovation of new oligomeric states that would have been overlooked from solely relying on existing structural databases. These findings are in agreement with the aforementioned hypothesis, such that the ancestral enzymes characterized in our study did occupy different oligomeric states over the phylogeny, to the extent that all three known form II assemblies (dimer, tetramer, and hexamer) were observed at different points. Future applications of this diversity-driven approach should help assess whether similar trajectories are found in other protein families, greatly increasing our understanding of the occurrence of oligomeric plasticity over evolutionary time.

It has been recently hypothesized that proteins increase in oligomeric complexity due to ratchet-like evolution mediated by hydrophobic interactions (*3*). Although there are examples of this, not all molecular complexes are formed and stabilized through hydrophobic patches, as we have demonstrated in this instance via the solvent-accessible polar interactions found in RuBisCO. The observed plasticity of form II RuBisCO illustrates the prevalence of oligomeric interconversion events in nature, demonstrating how evolutionary intermediaries can drift between two distinct assemblies before the evolutionary accumulation of additional mutations that result in commitment to either assembly. In the absence of strong selective pressures, the mutations that resulted in structural differentiation can be reversed, accordingly generating an overall reversion of oligomeric state. However, the presence of functional pressures can select for and entrench a particular oligomeric state, thus precluding any further reversion events. Conservation of interface residues reveals the mechanism by which these states exhibit these patterns of oligomerization, as a highly conserved set of interface residues may be found across all extant multimers, albeit bolstered with additional stabilizing contacts in clades demonstrating a strong commitment to a singular oligomeric state.

Our findings on form II RuBisCO provide the requisite evolutionary reference point to understand the evolutionary trajectory and structural basis of form I RuBisCO, the most abundant enzyme on our planet. Unlike all other forms of RuBisCO, the distinguishing feature of form I RuBisCO is its unique incorporation of small subunits to assemble its iconic heteromeric complex composed of eight large and eight small subunits. Form I RuBisCO likely underwent an early differentiation event from an ancestral dimeric state of all RuBisCO, which subsequently strongly entrenched the octameric core assembly with the acquisition of the small subunit. While the initial binding event between an ancestral octamer and a small subunit-like protein may have occurred with no tangible benefit conferred to either protein (i.e., via constructive neutral evolution), extant form I enzymes suffer from markedly decreased activity in the absence of their native small subunits, thus predicating overall activity on the hexadecameric assembly (*38*). However, form II RuBisCO do not demonstrate a noticeable trend relating oligomeric state to carboxylation activity, further suggesting that the function of form II RuBisCO is largely independent of its oligomeric state (fig. S11). Overall, the comparison of the two divergent evolutionary paths taken by form II versus form I RuBisCO provides a dichotomy in structural plasticity versus entrenchment, respectively. The structural plasticity of form II RuBisCO has resulted in a complex history of various oligomerizations, whereas the innovation and incorporation of the small subunit was the crux in the ratchet-like evolution that gave rise to the form I clade. The strict requirements of form I assembly for catalytic activity are not shared by form II, thus permitting the structural plasticity that enabled the innovation and maintenance of new oligomeric states. While we demonstrate in vitro retracing of the trajectory of RuBisCO oligomeric plasticity, future in vivo studies will be necessary to decipher the role of the observed phenomena in a biological context. Broadly, it remains unknown as to whether or not biochemical experiments accurately reflect the biological context in which assayed enzymes function in vivo. This is further obfuscated in the case of microbial RuBisCOs, wherein intracellular concentration and dynamics are poorly understood, and further exacerbated through the study of microbial RuBisCOs derived from metagenomes. This highlights the need for continued investigation of form II RuBisCOs; while prior research has primarily been dedicated to characterizing the behavior and levels of form I RuBisCO in plants (*10*), we presently lack the foundational knowledge necessary to understand and interpret the kinetic properties derived from form II–containing organisms and their respective enzymes (*16*).

The low-throughput nature of structural studies in combination with sparse phylogenetic sampling has left gaps in our understanding of protein evolution at the molecular level; thus, most of our knowledge of oligomeric plasticity largely stems from single to few representatives. Diversity-driven studies will help shed light on the complex range of evolutionary paths and disparate oligomeric states that can be observed within individual protein subfamilies. Our results demonstrate how quaternary structure may be inherently malleable until functional roles entrench specific oligomeric states, thus allowing proteins to sample and explore not only sequence space but also disparate oligomeric states. Notably, we also show how changes in quaternary structure may also contribute to the tuning of enzyme kinetics, providing a potential avenue of selective pressure on oligomeric state. Given the central role oligomeric state may play in many proteins, it remains to be shown how prevalent quaternary structural plasticity is across other protein families, as it may represent a nuanced, yet important, contributor shaping the evolution of protein structure and function.

## MATERIALS AND METHODS

### Phylogenetic analyses

Form II and II/III amino acid sequences were originally compiled from UniProtKB (www.uniprot.org/) using the search functions “rubisco” under protein name and “cbbM” under gene name. The query results were assessed for inclusion on the basis of sequence length and annotated oligomeric state. Form II/III sequences were included on the basis of high-sequence homology (>70%) to *Methanococcoides burtonii* RuBisCO. The resulting UniProtKB sequence library was combined with the amino acid sequence library studied in Davidi *et al.* (*16*). RuBisCO sequences were then dereplicated at 97% amino acid identity using CD-Hit (*39*).

Sequences from the final library were aligned with MAFFT using default parameters (https://mafft.cbrc.jp/alignment/server/) (*40*). Columns with >90% gaps were removed using TrimAI (http://phylemon2.bioinfo.cipf.es/). The evolutionary model most appropriate for constructing a phylogenetic tree was determined using Prottest 3.0 (*41*). A maximum likelihood phylogenetic tree was constructed using RAxML-HPC BlackBox (v. 8.2.12) as implemented on cipres.org (default parameters with WAG model) with form II/III sequences as the outgroup. The BOOSTER method was subsequently used to calculate the bootstrap branch support for the resulting phylogenetic tree (https://booster.pasteur.fr/) using “RAxML_bestTree” as the input reference tree and “RAxML_bootstrap” as the input bootstrap tree. All files used to create the phylogenetic trees are included on figshare.

### Ancestral sequence reconstruction

Ancestral sequence reconstruction was performed with FastML v3.1 (http://fastml.tau.ac.il/) using the RuBisCO multiple sequence alignment and associated RAxML phylogenetic tree. Default parameters were selected, including branch length optimization, use of gamma distribution, indel reconstruction, and joint reconstruction computation. The sequences of the marginal reconstruction (including ancestral reconstruction of indels) were initially inferred using an indel cutoff of 0.2, 0.4, 0.6, 0.8, and 1.0. Amino acid sequence motifs and gaps from the form II clade were most similar to the ancestral sequences constructed with either an indel cutoff value of 0.6 or 0.8, both of which produced near identical results. An indel cutoff of 0.6 was chosen for the final ancestral sequence reconstruction. All files used to create inferred ancestral sequences are included on figshare.

### Relative amino acid evolutionary rate analysis

The relative evolutionary rates of amino acid residues found in hexameric form II RuBisCO were computed with Rate4Site v2.01 (www.tau.ac.il/~itaymay/cp/rate4site.html) (*42*). First, the amino acid sequences for hexameric form II RuBisCO (including those identified in this study) were aligned with MAFFT using default parameters (https://mafft.cbrc.jp/alignment/server/) (*40*). A maximum likelihood phylogenetic tree was subsequently constructed using RAxML-HPC BlackBox (v. 8.2.12) as implemented on cipres.org (default parameters with WAG model). The MSA and associated phylogenetic tree were then used as input for Rate4Site to calculate the relative conservation score for each site in the MSA.

### Expression and purification of RuBisCO

Heterologously expressed RuBisCO were purified in a manner similar to previously described methods (*12*, *16*). BL21 DE3 Star competent *Escherichia coli* cells (MacroLab, Berkeley, USA) were transformed with a pET28 plasmid containing the corresponding His_14_-bdSUMO–tagged RuBisCO sequence. Cells were grown at 37°C to an optical density at 600 nm of ~0.6 to 0.8, followed by induction with 1 mM isopropyl-β-d-thiogalactopyranoside and further incubation overnight at 16°C. Cell cultures were then pelleted, resuspended in lysis buffer (pH 8.0; 50 mM sodium phosphate, 300 mM NaCl, 10 mM imidazole, 5% glycerol, and 2 mM MgCl_2_), and subjected to a freeze-thaw cycle. Thawed cells were then lysed using an EmulsiFlex-C3 (AVESTIN Inc., Ottawa, Canada). Lysate was clarified by centrifugation at 15,000*g*, and soluble fractions were 0.44 μm, filtered before application to preequilibrated Ni–nitrilotriacetic acid (NTA) resin for batch binding. Columns were washed twice, first with a 25 mM imidazole wash buffer (20 mM sodium phosphate, 300 mM NaCl, 25 mM imidazole, and 10% glycerol), followed by a 50 mM imidazole wash buffer (20 mM sodium phosphate, 300 mM NaCl, 50 mM imidazole, and 10% glycerol). The column was then resuspended in SUMOlase buffer [pH 8.0; 20 mM Hepes-OH, 100 mM NaCl, 1 mM dithiothreitol (DTT), 15 mM imidazole, and 20 mM MgCl_2_], and purified bdSENP1 was added and incubated overnight to facilitate tag cleavage (*12*, *43*). Flow-through from the cleavage reaction was collected and analyzed by SDS–polyacrylamide gel electrophoresis (PAGE) for purity.

### Size exclusion chromatography coupled small-angle x-ray scattering with in-line multiangle light scattering experiments

RuBisCO was purified as described above and concentrated to 2 to 5 mg/ml. Concentrated RuBisCO was then activated with an excess of NaHCO_3_ before sample analysis. SEC-SAXS-MALS data were collected at the Advanced Light Source beamline 12.3.1 at Lawrence Berkeley National Lab (Berkeley, CA, USA) (*44*). The x-ray wavelength was set at λ = 1.24 Å, and the sample-to-detector distance was 2075 mm resulting in scattering vectors (*q*) ranging from 0.01 to 0.46 Å^−1^. The scattering vector is defined as *q* = 4πsinθ/λ, where 2θ is the scattering angle. Data were collected using a Pilatus 3X 2M Detector (DECTRIS, Baden, Switzerland). Normalization and integration of each image were processed as previously described (*17*). SEC was performed using the 1290 Infinity High-Performance Liquid Chromatography System (Agilent, Santa Clara, CA) coupled to a Shodex KW-803 column (Showa Denko, Tokyo, Japan). The column was equilibrated with a running buffer [20 mM Hepes-OH (pH 8.0), 300 mM NaCl, 10 mM MgCl_2_, and 10 mM NaHCO_3_] at a flow rate of 0.65 ml/min. Ninety to 100 μl of sample was separated by SEC, and the elution was monitored at 280 and 260 nm by an in-line variable wavelength detector (Agilent, Santa Clara, CA). MALS experiments were performed using an in-line 18-angle DAWN HELEOS II light scattering detector connected in tandem to an Optilab differential refractive index (dRI) detector (Wyatt Technology, Goleta, CA). System normalization and calibration were performed with bovine serum albumin using a 50-μl sample at 7 mg/ml in the same running buffer. The light scattering experiments were used to determine the molecular weight across the principal peaks in the SEC analysis (fig. S10). Ultraviolet, MALS, and dRI data were analyzed using Wyatt Astra 7 software to monitor the homogeneity of the sample across the elution peak complementary to the SEC-SAXS signal validation. A purpose-built SAXS flow cell was connected in-line immediately following the complementary spectroscopic techniques and 2-s x-ray exposures were collected continuously over the 25-min elution. The SAXS frames recorded before the protein elution peak were used to subtract all other frames. The subtracted frames were investigated by radius of gyration (Rg) derived by the Guinier approximation, *I*(*q*) = *I*(0) exp(−*q*^2^Rg2/3) with the limits *q*Rg < 1.5. The elution peak was mapped by comparing integral ratios to background and Rg relative to the recorded frame using the program ScÅtter (*45*). Uniform Rg values across an elution peak represent a homogenous assembly and were merged to reduce noise in the curve. Final merged SAXS profiles (Figs. 3 and 4 and figs. S2 and S3) were used for further analysis including the Guinier plot that determined aggregation-free state. The experimental SAXS profiles were then compared to theoretical scattering curves generated from atomistic models of *R. rubrum* [Protein Data Bank (PDB): 5RUB] (fig. S2), the *S. caldicuralii* tetramer (fig. S2), hexameric and dimeric *Gallionella* sp. states (Fig. 3C and fig. S2), modified tetrameric form I enzyme (fig. S4, A and B), and engineered *I. peregrinum* enzyme (Fig. 4C) using FoXS (*46*, *47*).

### Crystallization and structural determination of RuBisCO

Ni-NTA–purified RuBisCO were further subject to anion exchange chromatography on a MonoQ 10/100 GL column and eluted by a linear NaCl gradient from 5 mM to 1 M. Fractions were analyzed by SDS-PAGE, followed by concentration and SEC on a Superose 6 Increase 10/300 GL, in a final buffer containing 100 mM Hepes (pH 8), 100 mM NaCl, 25 mM MgCl_2_, 5 mM NaHCO_3_, and 1 mM DTT. Samples were activated as previously described before incubation with a tenfold molar excess of previously synthesized 2-carboxyarabinitol 1,5-bisphosphate (CABP) (*48*).

The tetrameric *S. caldicuralii* RuBisCO and the hexameric BRH_c57 RuBisCO were screened against the following crystallization screens: MCSG-1 (Anatrace); Crystal Screen, SaltRx, PEG/Ion, Index, and PEGRx (Hampton Research); and Berkeley Screen (*49*). Crystals of the *S. caldicuralii* RuBisCO were found in 0.05 M citric acid, 0.05 M bis-tris propane (pH 5.0) and 16% polyethylene glycol (PEG) 3350. Crystals of the BRH_c57 RuBisCO were found in 0.2 M magnesium formate (pH 5.9) and 20% PEG 3350. Crystals from both enzymes were then placed in a reservoir solution containing 20% (v/v) glycerol and flash-cooled in liquid nitrogen.

The x-ray dataset for the *S. caldicuralii* RuBisCO was collected at the Berkeley Center for Structural Biology beamline 5.0.2 at the Advanced Light Source at Lawrence Berkeley National Laboratory, and the BRH_c57 dataset was collected at the Frontier Microfocusing Macromolecular Crystallography (FMX) beamline at the National Synchrotron Light Source II at Brookhaven National Laboratory. The diffraction data were processed using the program Xia2 (*50*). The crystal structures of *S. caldicuralii* and BRH_c57 were solved using molecular replacement with the program PHASER (*51*). The atomic positions obtained from the molecular replacement were used to initiate model building using phenix.autobuild within the Phenix suite (*52*, *53*). Structure refinement was performed using the phenix.refine program (*54*). Manual rebuilding was done using COOT (*55*). Root mean square deviation differences from ideal geometries for bond lengths, angles, and dihedrals were calculated with Phenix (*53*). The stereochemical quality of the final models of *S. caldicuralii* and BRH_c57 was assessed by the program MOLPROBITY (*56*). A summary of crystal parameters, data collection, and refinement statistics can be found in table S2. Structures and coordinates for *S. caldicuralii* and BRH_c57 RuBisCO can be found in the PDB under accession IDs 7T1C and 7T1J, respectively.

### RuBisCO activity assays

Purified RuBisCO was used to determine catalytic properties as described previously (*57*), with some alterations to protein desalting and activation: Concentrated protein aliquots were first diluted with activation mix containing 100 mM bicine-NaOH (pH 8.0), 20 mM MgCl_2_, 10 mM NaHCO_3_, and 1% (v/v) plant protease inhibitor cocktail (Sigma-Aldrich, UK). RuBisCO was then activated on ice for 20 min before being used in ^14^CO_2_ consumption assays at 25°C with CO_2_ concentrations of 50, 100, 200, 300, and 400 μM. To determine *K*_O_, these CO_2_ concentrations were combined with concentrations of 0, 21, 40, or 70% (v/v) O_2_. *k*_cat_^O^ was calculated from measured parameters using the equation *S*_C/O_ = (*V*_C_*/K*_C_)/(*V*_O_*/K*_O_). *k*_cat_^C^ was determined using measurements with 0% O_2_. An aliquot of the activated protein was used for determination of RuBisCO active sites via ^14^C-CABP binding using the method of Sharwood *et al.* (*58*). RuBisCO specificity was determined using the method of Parry *et al.* (*59*). Measurements using *Triticum aestivum* (bread wheat) RuBisCO were used for normalization as previously described, with a p*K*_a_ of 6.11 used for calculations (where *K*_a_ is the acid dissociation constant).

### Protein contacts atlas analyses

Interface residues of the *S. caldicuralii* tetramer (PDB: 7T1C) and the *Gallionella* sp. hexamer (PDB: 5C2C) were identified using Protein Contacts Atlas (*22*).

### Site-directed mutagenesis experiments

Site-directed mutagenesis was conducted using an Agilent QuikChange Multi kit using primers designed by the Agilent QuikChange Primer Design tool (www.agilent.com/store/primerDesignProgram.jsp). Mutant RuBisCO was expressed and purified as previously described.

### Homology modeling

RosettaCM was used to prepare a homology model for the input structure of the dimeric *I. peregrinum* enzyme (*60*). MUSCLE was used for global sequence alignment during homology modeling (*61*). Expanded sampling on side-chain chi angles resolved dimer-dimer interfacial interaction more accurately using level 4 Rosetta rotamer libraries (*62*). The flags and xml script used in homology modeling are available in the Supplementary Materials.

### Symmetry definition

The symmetry definition was produced from make_symmdef_file.pl in Rosetta using the BRH_c57 structure as the input, “perl make_symmdef_file.pl -m NCS -p _49.pdb -a A -i C B -r 12 > _49.symm.”

### Mutant selection

Mutation sites were identified by locating interfacial residues where BRH_c57 and *I. peregrinum* differ in protein sequence. Residues were defined as interfacial if (i) they were within 5.5 Å of the opposite dimeric subunit or (ii) the side chain points to the opposite dimeric subunit within 9 Å. The mutation sites were manually screened, and seven sites were picked. All 128 combinations, each identified as a mutant, were modeled in silico as described below.

### In silico mutation

In silico mutagenesis was performed on all 128 mutants. Monomeric RuBisCO structure was first extracted from the *I. peregrinum* homology model and then applied with hexameric symmetry from the BRH_c57 structure (PDB: 7T1J). For each mutant, the residue(s) was mutated, and the surroundings within a 12-Å sphere of any mutation site were relaxed using the FastRelax protocol in Rosetta with level 4 rotamer libraries (*62*–*65*). For each mutant, the structure was independently sampled 50 times and then ranked by its total energy (total score). The five samples with the lowest total energy were assessed with the number of dimer-dimer hydrogen bonds made, defined by a distance cutoff of 3.6 Å. Upon manual inspection, mutants with the most hydrogen bonds were picked for experimental verification.

### Other software

Multiple sequence alignments were generated using MAFFT and visualized with ESPript 3.0 (*40*, *66*). Phylogenetic trees were visualized using Interactive Tree of Life v5 (*67*). UCSF ChimeraX was used for visualization of protein models and preparation of manuscript figures (*68*, *69*).

## Supplementary Materials

### This PDF file includes:

Figs. S1 to S11 Tables S1 and S2 References

## Notes

View/request a protocol for this paper from *Bio-protocol*.
