# Supplementary material for: Structural plasticity enables evolution and innovation of RuBisCO assemblies
Source: Sci Adv. 2022 Aug 26;8(34):eadc9440. doi: 10.1126/sciadv.adc9440 (PMC9417184; doi:10.1126/sciadv.adc9440)
Supplement: Supplementary file 1 — Figs. S1 to S11 Tables S1 and S2 References [file sciadv.adc9440_sm.pdf]

Science Advances
AAAS

Supplementary Materials for

Structural plasticity enables evolution and innovation of RuBisCO assemblies

Albert K. Liu *et al.*

Corresponding author: Patrick M. Shih, pmshih@berkeley.edu

Sci. Adv. **8**, eadc9440 (2022)
DOI: 10.1126/sciadv.adc9440

The PDF file includes:

Figs. S1 to S11
Tables S1 and S2
Legend for data file S1
References

Other Supplementary Material for this manuscript includes the following:

Data file S1

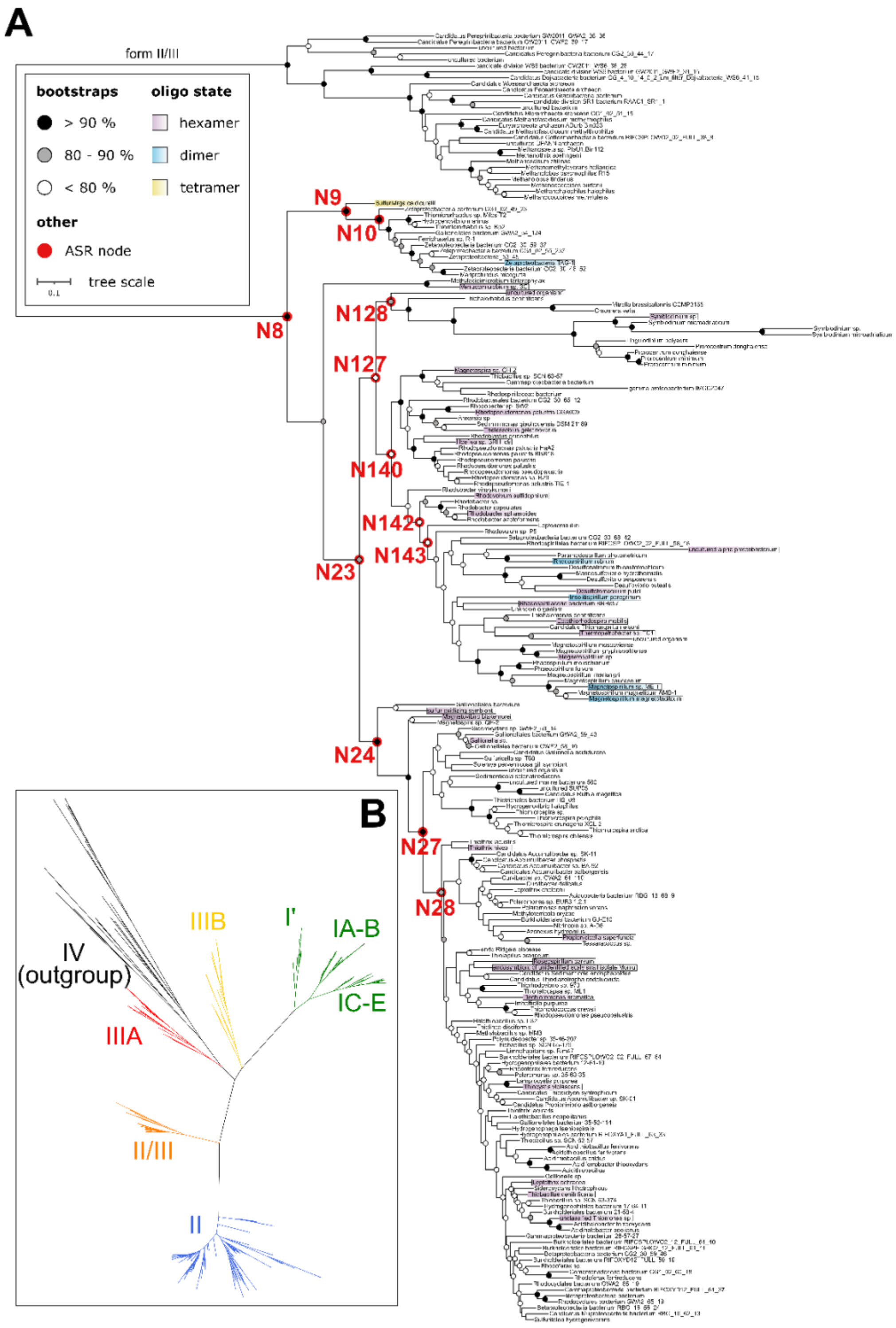

Fig. S1: Expanded phylogenetic tree of form II rubisco and all rubisco forms. (A) Form II rubisco phylogeny shown with species names at tips. Known oligomeric states indicated in color. Ancestral sequence nodes highlighted and labeled in red. **(B)** Phylogenetic tree of all rubisco forms. Clades color-coded with accompanying names.

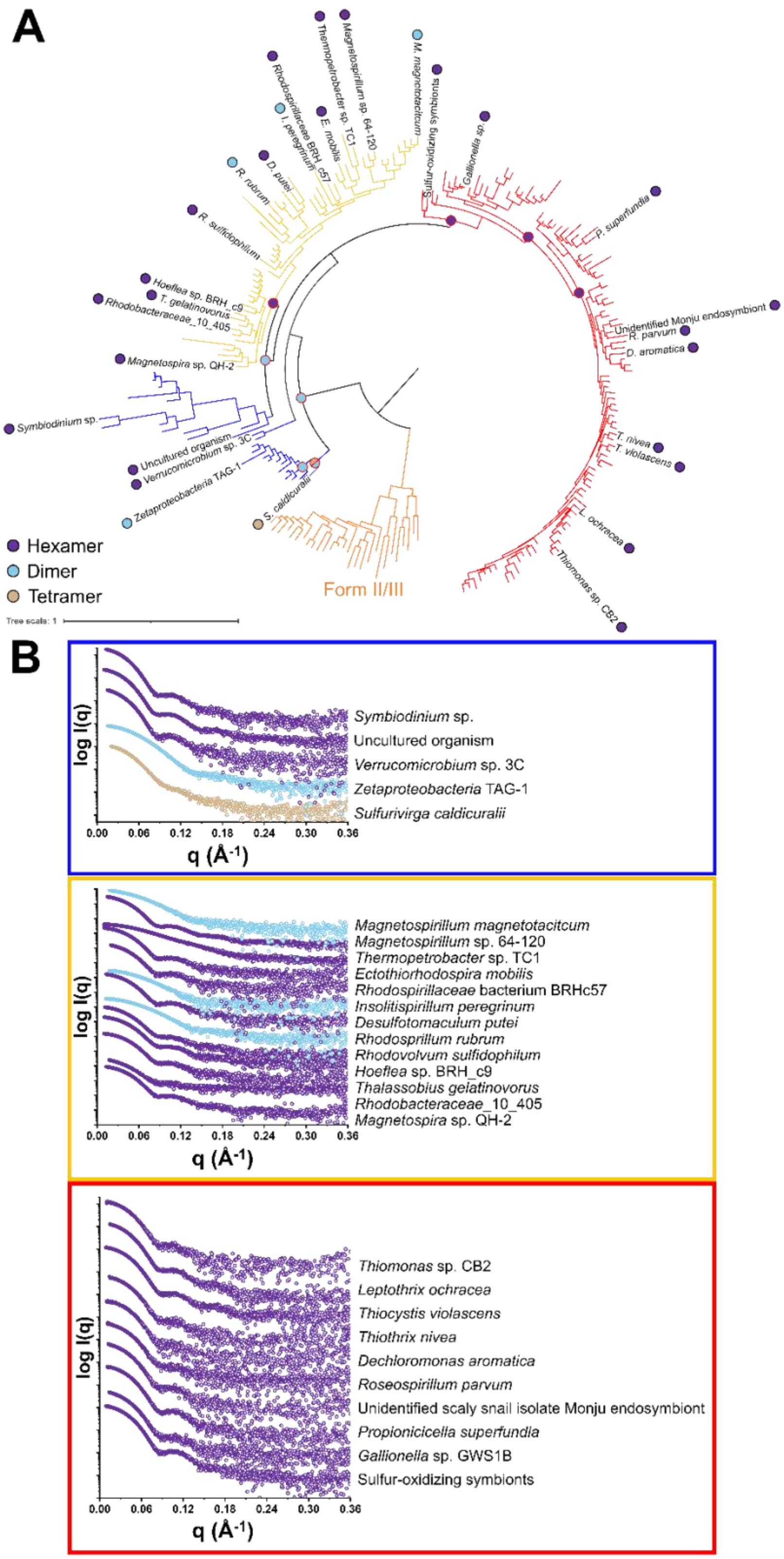

Fig. S2: SAXS curves of 28 analyzed form II rubisco. (A) Color-coded phylogenetic tree (adapted from Fig. 1). **(B)** SAXS curves of samples in corresponding color-coded regions.

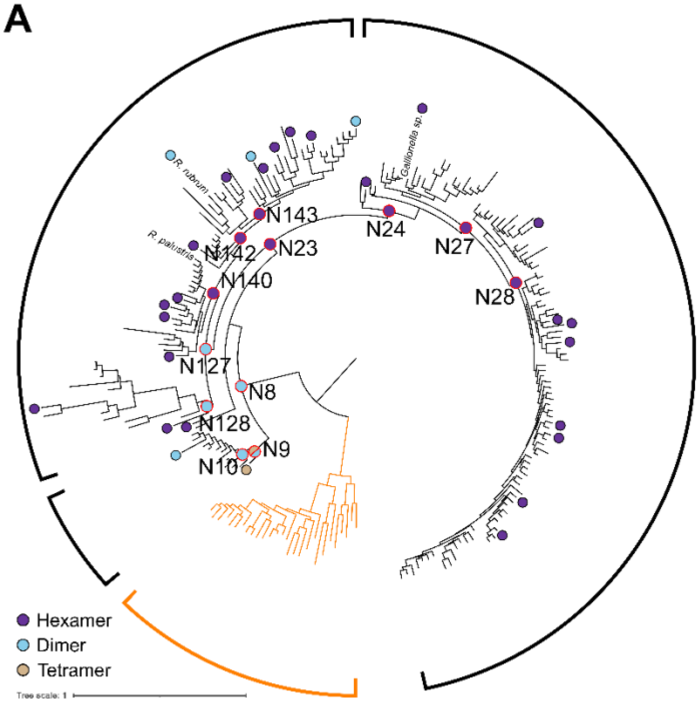

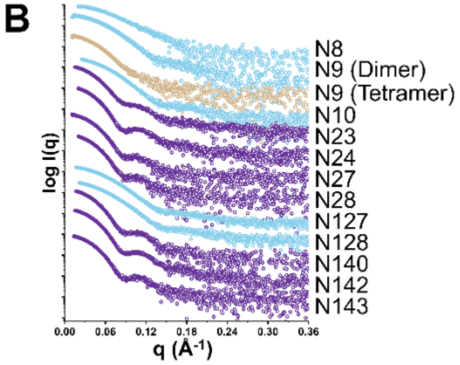

C

Sample:	Mw (kDa)	R(h) (nm)	Oligomeric state:
N8	102.5	3.401	Dimer
N9 (Dimer)	110.7	3.712	Dimer
N9 (Tetramer)	216.3	4.804	Tetramer
N10	99.44	3.874	Dimer
N23	281.3	5.624	Hexamer
N24	292.5	5.751	Hexamer
N27	326.4	5.662	Hexamer
N28	328.7	5.585	Hexamer
N127	106	3.849	Dimer
N128	110	4.218	Dimer
N140	315	5.827	Hexamer
N142	312	5.78	Hexamer
N143	315.9	5.899	Hexamer

Fig. S3: SEC-SAXS-MALS analysis of ancestral form II enzymes. (A) Form II phylogenetic tree with indicated locations and names of ancestral nodes. **(B)** SAXS curves of characterized ancestral enzymes. **(C)** Collected MALS values with measured molecular weights, radii of hydration, and oligomeric states.

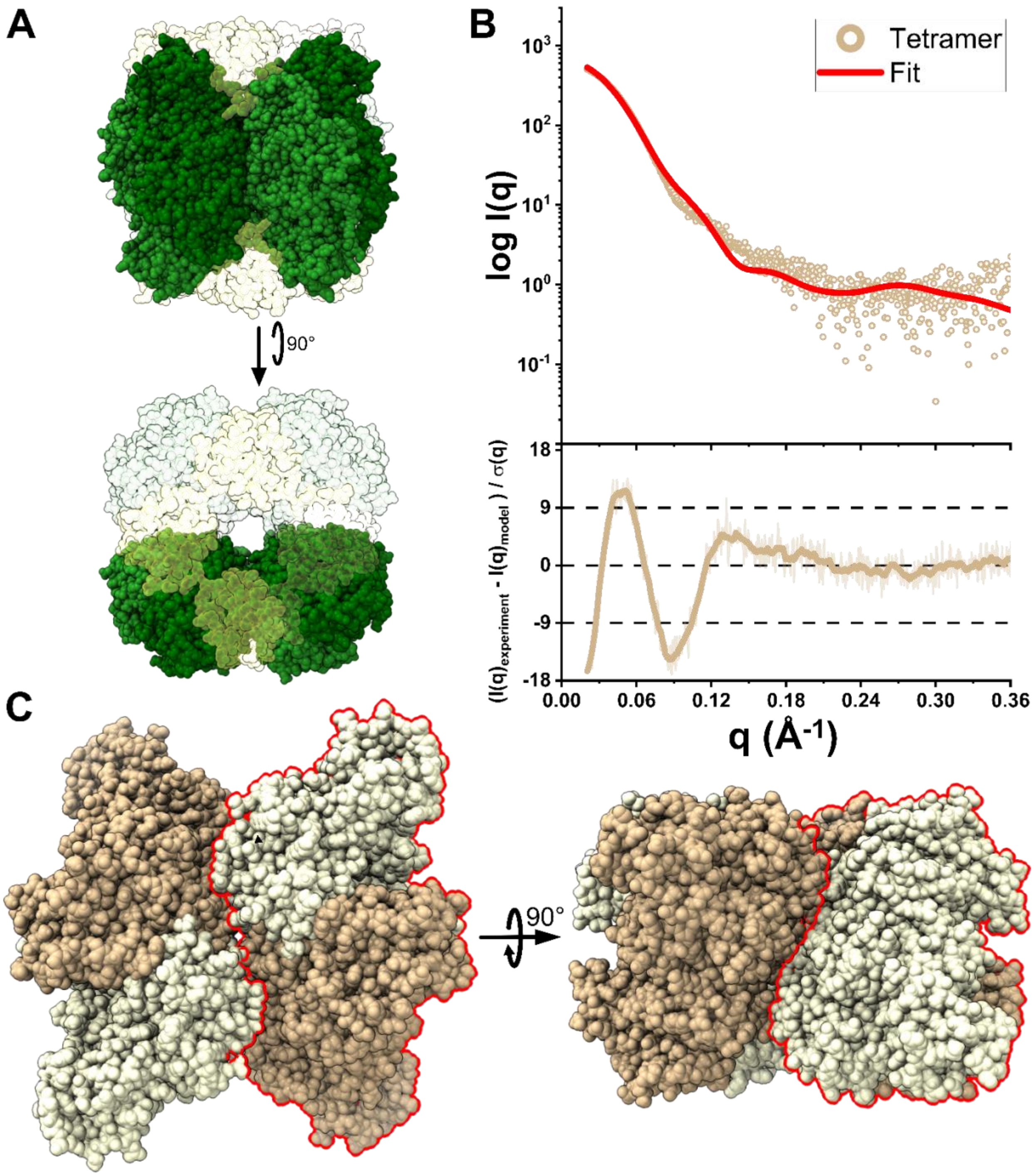

Fig. S4: Novel tetrameric rubisco structurally distinct from other rubisco assemblies. (A) Theoretical generation of tetrameric assembly from form I rubisco. Positions of two dimers indicated within hexadecamer (PDB: 1RBL). **(B)** SAXS curve of *Sc* tetramer fit against form I tetrameric assembly from (A). Fit-residuals shown below. **(C)** Position of base dimer within *Sc* tetramer. Single dimer outlined in red.

A

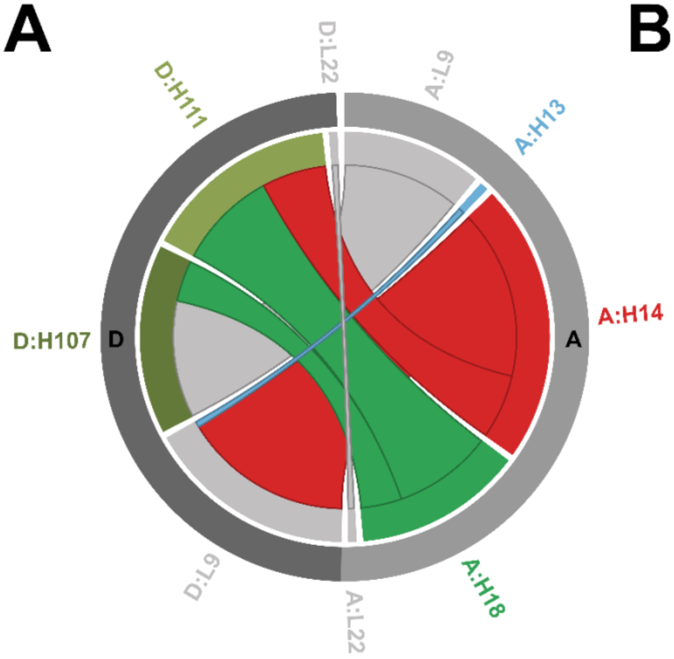

B

	I100	D101	K103	E250	N254	H284	Y285
I100				2			
D101					1		
K103					6		
E250	2					**2**	
N254		1	6				
H284				2			3
Y285							

Fig. S5: Interface residue identification of *S. caldicuralii* tetramer. (A) Protein Contacts Atlas chord plot illustrating interaction network between chains A and D of structure. **(B)** Heat map of unique side chain-side chain interactions between interface residues.

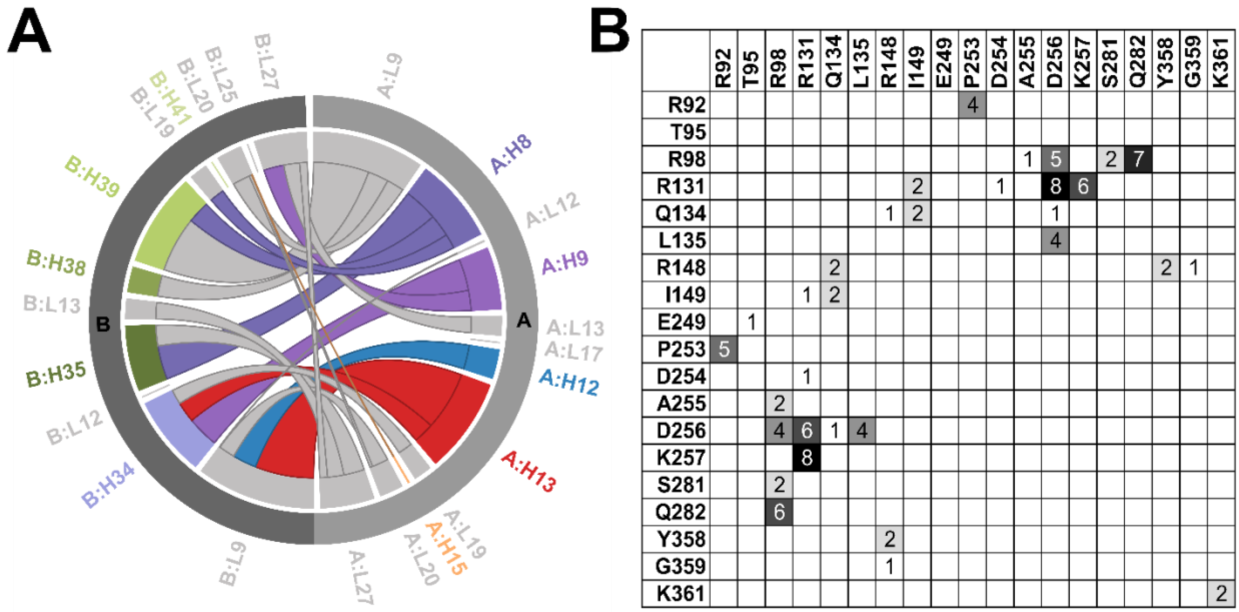

B

	R92	T95	R98	R131	Q134	L135	R148	I149	E249	P253	D254	A255	D256	K257	S281	Q282	Y358	G359	K361
**R92**										4									
**T95**																			
**R98**												1	5		2	7			
**R131**								2			1		8	6					
**Q134**							1	2					1						
**L135**													4						
**R148**					2												2	1	
**I149**				1	2														
**E249**		1																	
**P253**	5																		
**D254**				1															
**A255**			2																
**D256**			4	6	1	4													
**K257**				8															
**S281**			2																
**Q282**			6																
**Y358**							2												
**G359**							1												
**K361**																			2

Figure S6: Interface residue identification of *Gallionella* sp. hexamer. (A) Protein Contacts Atlas chord plot illustrating interaction network between secondary structures on chains A and B of 5C2C structure. **(B)** Heat map of unique side chain-side chain interactions between interface residues.

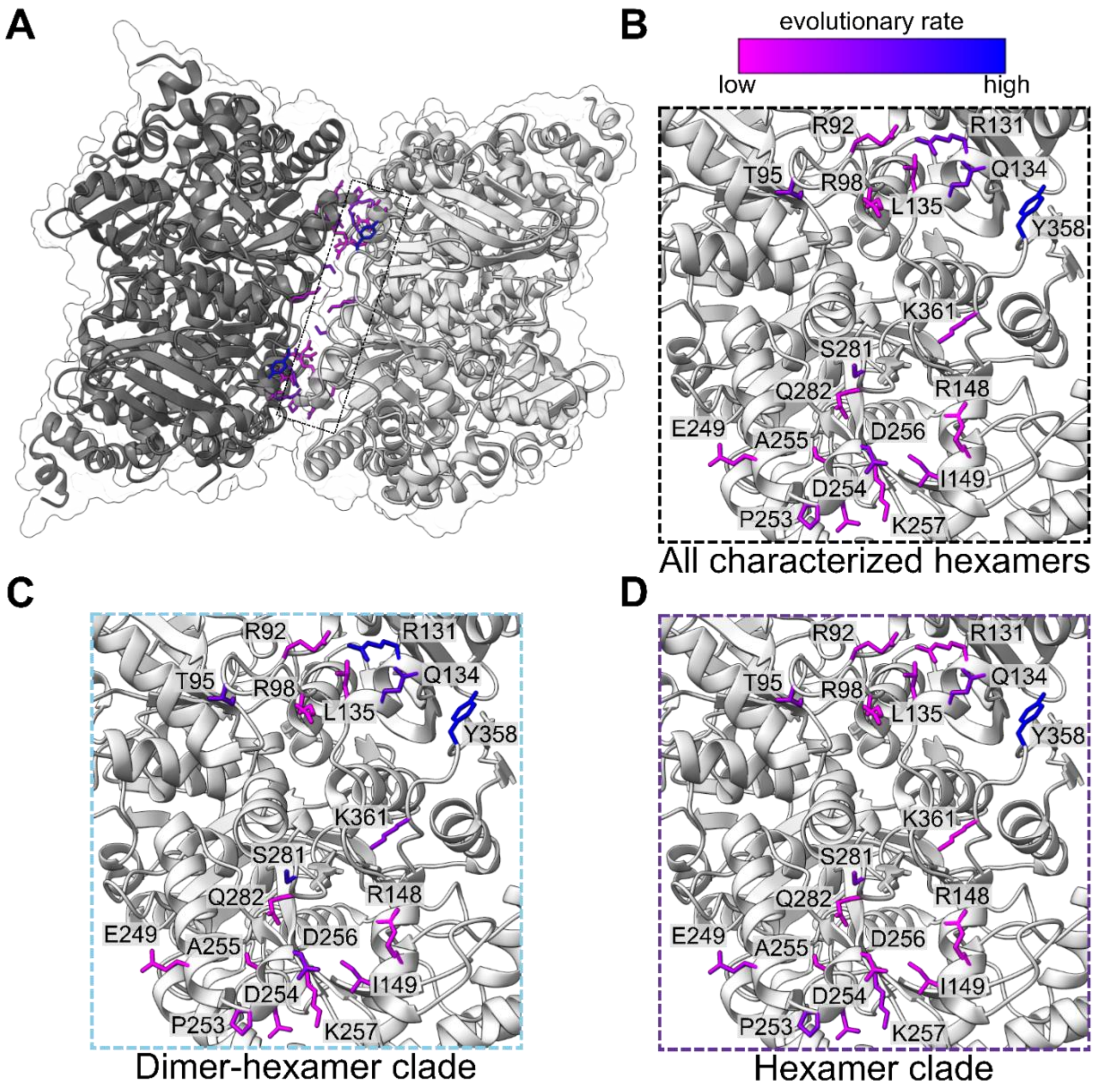

Figure S7: Sequence conservation analysis of hexameric *Gallionella* sp. interface. (A) Location of interface residues on *Gallionella* sp. crystal structure (PDB: 5C2C). **(B)** Conservation of residues as determined from all characterized hexamers. Low evolutionary rate (highly conserved amino acid position) in magenta, scaling to high evolutionary rate (more variable amino acid position) in blue. Score values utilized for color-coding generated according to (42)**. (C)** Residue conservation analysis conducted exclusively with hexamers in dimer-hexamer clade. **(D)** Residue conservation analysis conducted exclusively with hexamers from hexamer clade.

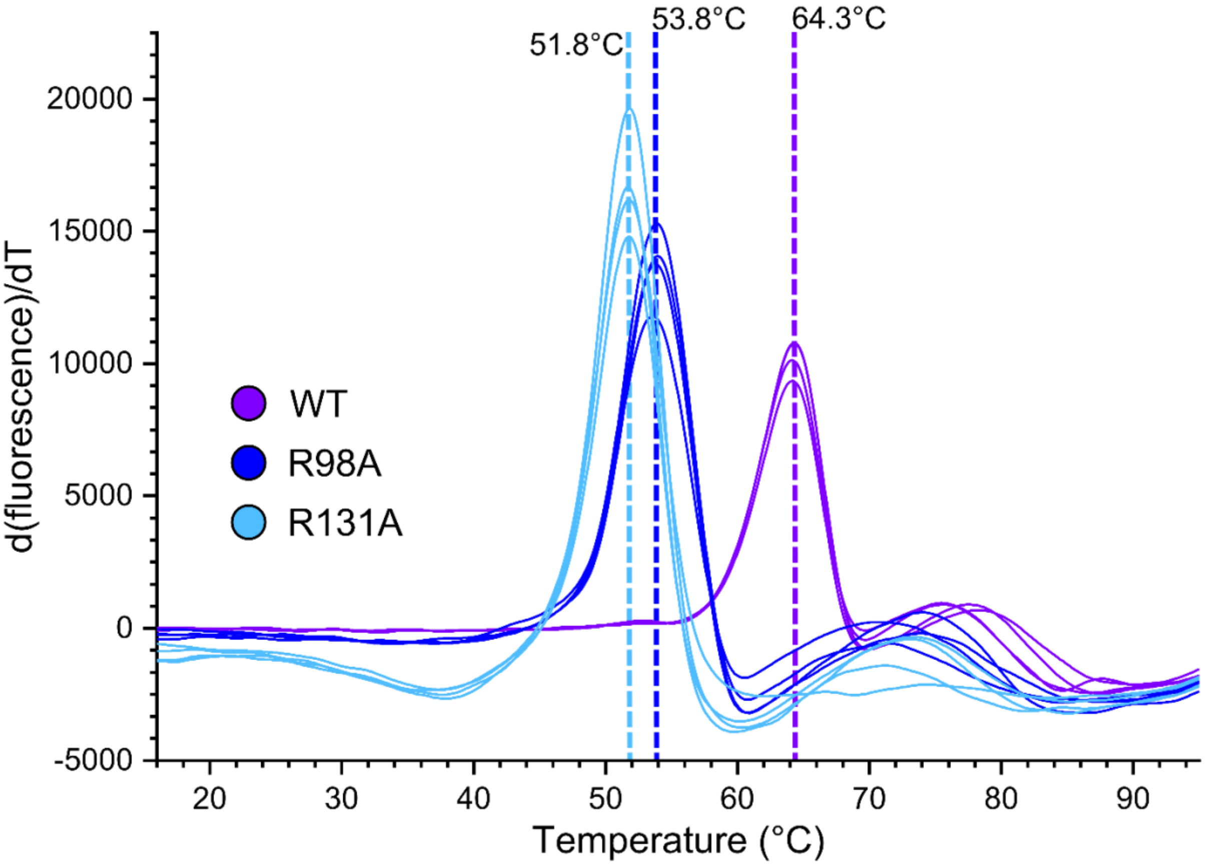

Figure S8: Protein Thermal Shift assay melt curves for WT *Gallionella* sp. hexamer and R98A, R131A dimers. Reported T_m values represent the average from four technical replicates.

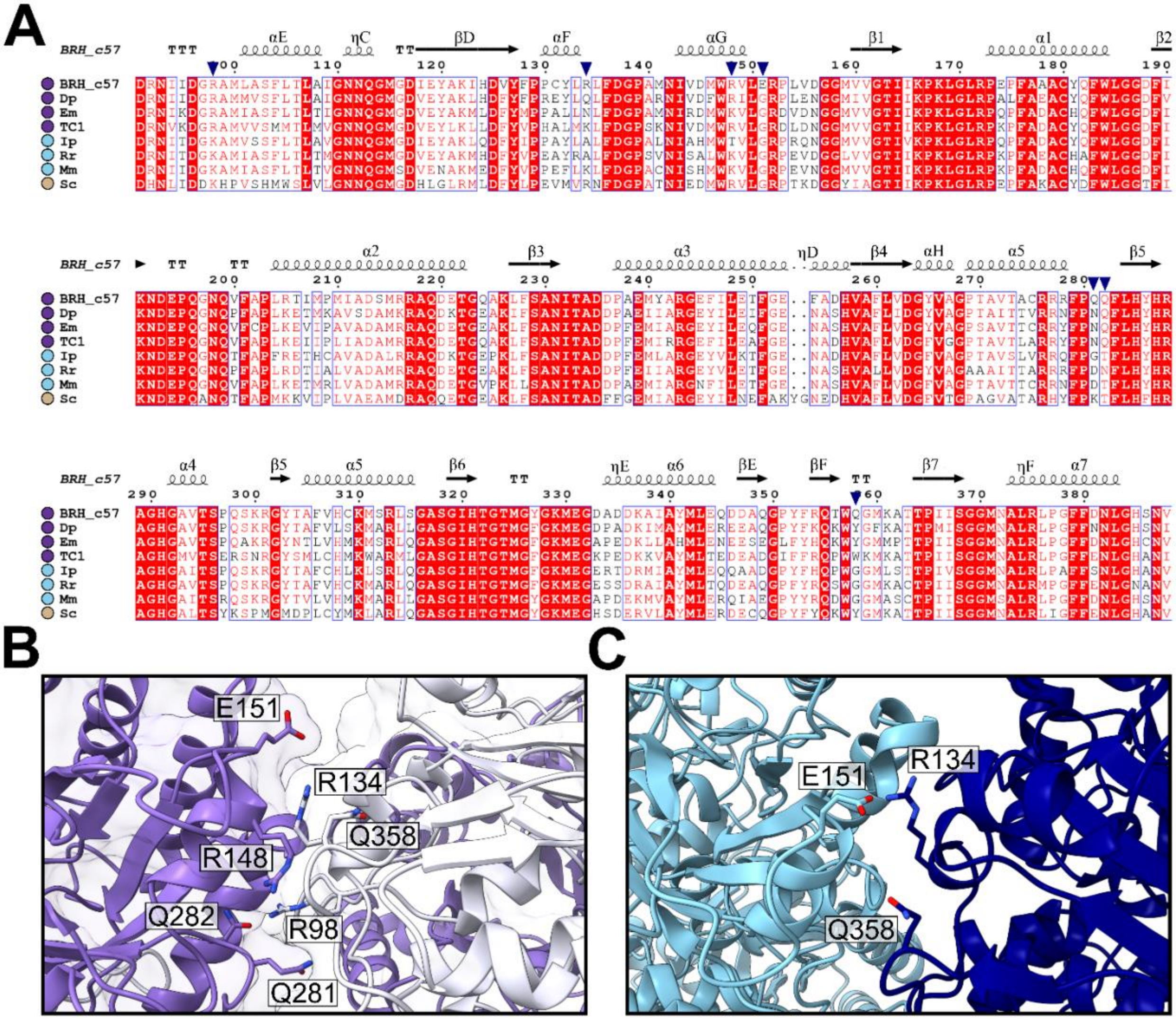

Figure S9: Identification and selection of hexameric residues for engineering. (A) Truncated alignment of representative hexamers, dimers, and tetramer. Oligomeric state indicated by circles to left of species name (hexamers; purple, dimers; blue, tetramer; tan). Interface residues indicated by inverted blue triangles. Species listed from top to bottom: *Rhodospirillaceae* bacterium BRH_c57, *Desulfotomaculum putei*, *Ectothiorhodospira mobilis*, *Thermopetrobacter* sp. TC1, *Insolitispirillum peregrinum*, *Rhodospirillum rubrum*, *Magnetospirillum magnetotacitcum*, *Sulfurivirga caldicuralii*. Secondary structure labels assigned according to rubisco nomenclature from (70). **(B)** Dimer-dimer interface cutaway of BRH_c57 crystal structure. Residues indicated. **(C)** Novel inter-dimer interaction formed in hexameric *Ip* mutant.

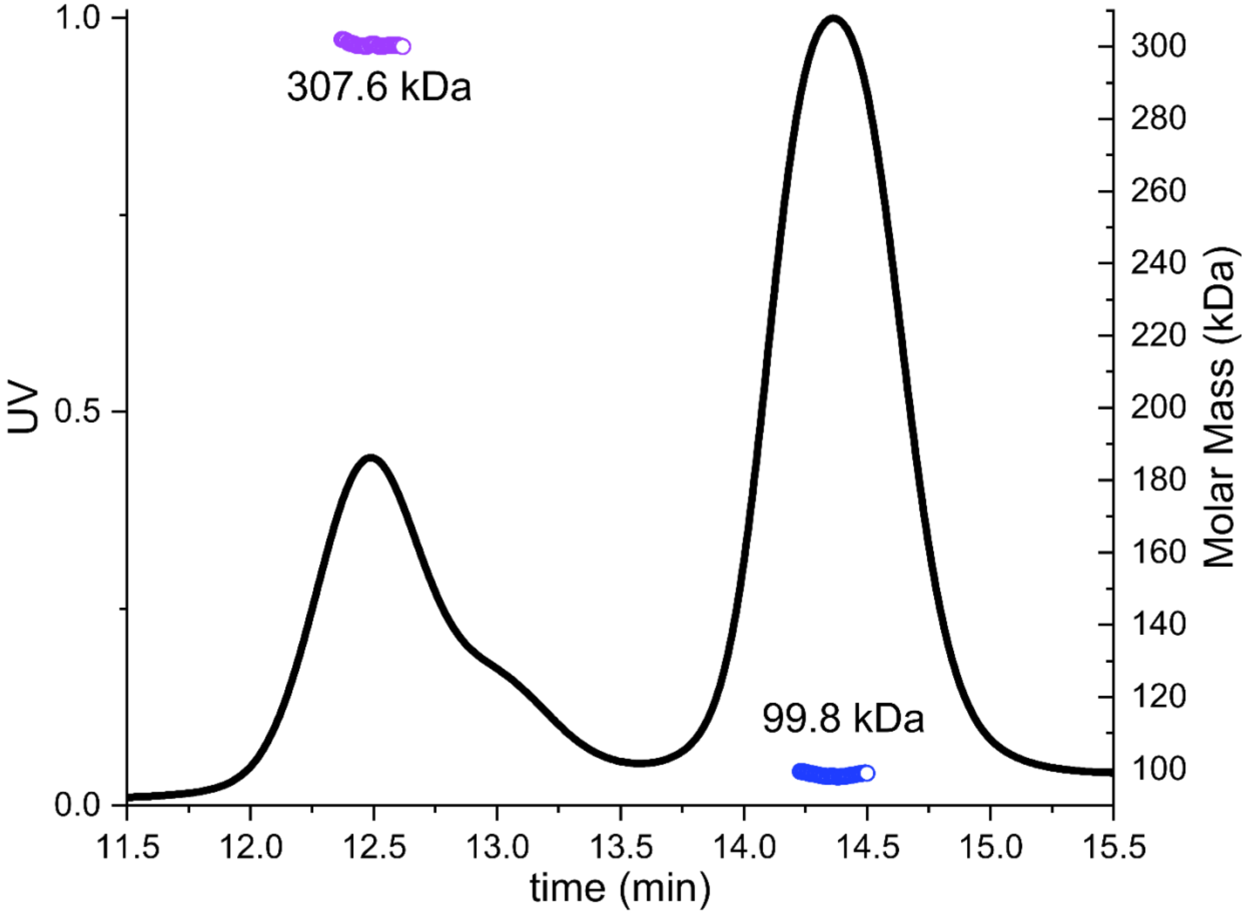

Figure S10: SEC-MALS chromatogram of engineered 2-to-6 sample. Molecular weight of each species indicated with each peak.

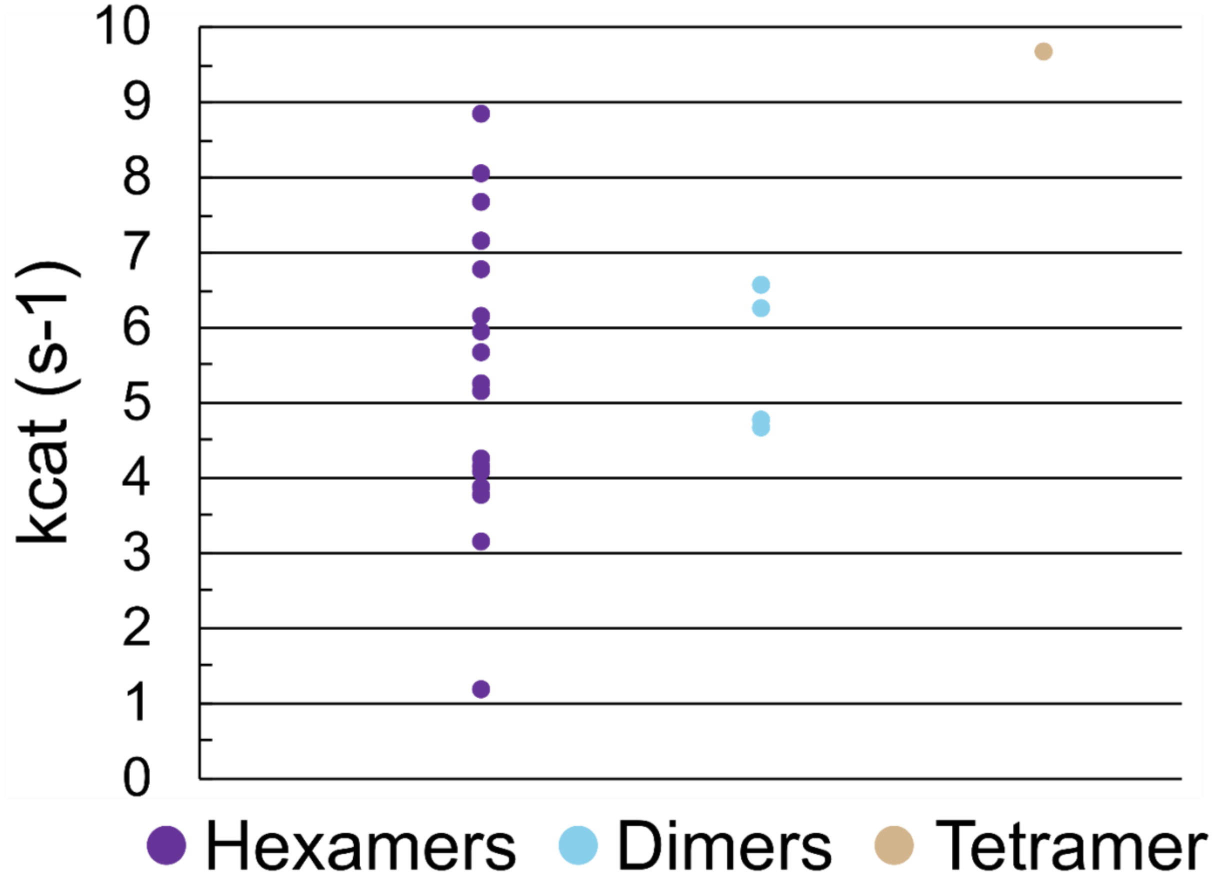

Figure S11: Spectroscopic k_{cat}^{C} values of extant form II enzymes characterized in this study. k_{cat}^{C} values from (12).

Table S1: MALS-determined molecular weights and radius of hydration values for 28 characterized form II rubisco.

Sample:	Mw (kDa)	R(h) (nm)	Oligomeric state:
Thiocystis violascens	325.8	5.375	Hexamer
Uncultured organism	332.4	5.645	Hexamer
Leptothrix ochracea	349.4	5.905	Hexamer
Zetaproteobacteria TAG-1	107.2	3.462	Dimer
Thalassobius gelatinovorus	313.2	5.35	Hexamer
Magnetospirillum magnetotacitcum	106	3.727	Dimer
Rhodovolvum sulfidophilum	300.5	5.809	Hexamer
Propionicicella superfundia	317.1	5.25	Hexamer
Thiothrix nivea	314.3	5.404	Hexamer
Verrucomicrobium sp. 3C	319.7	5.616	Hexamer
Thermopetrobacter sp. TC1	314.2	4.86	Hexamer
Symbiodinium sp.	302.4	5.334	Hexamer
Unidentified scaly snail isolate Monju endosymbiont	302.4	5.636	Hexamer
Magnetospira sp. QH-2	299.1	6.531	Hexamer
Magnetospirillum sp. 64-120	325	5.777	Hexamer

Desulfotomaculum putei	324.8	6	Hexamer
Hoeflea sp. BRH_c9	313.2	5.451	Hexamer
Thiomonas sp. CB2	313.7	4.693	Hexamer
Sulfur-oxidizing symbionts	299.5	5.712	Hexamer
Dechloromonas aromatica	349.2	6.561	Hexamer
Ectothiorhodospira mobilis	311.7	5.491	Hexamer
Roseospirillum parvum	337.5	6.313	Hexamer
Gallionella sp. GWS1B	314.3	5.45	Hexamer
Insolitispirillum peregrinum	101	2.854	Dimer
*Rhodobacteraceae*_10_405	274	3.273	Hexamer
Rhodospirillum rubrum	100.8	3.352	Dimer
Rhodospirillaceae bacterium BRHc57	368.3	5.703	Hexamer
Sulfurivirga caldicuralii	218.3	4.896	Tetramer

Table S2: Statistics for data collection and refinement of *Sc* and BRH_c57 rubisco.

	Sc (L_4)	BRH_c57 (L_6)
Data collection		
Wavelength (Å)	1.000	0.97936
Resolution range (Å)	42.94 – 1.73 (1.79 – 1.73)	29.52 – 1.96 (2.03 – 1.96)
Detector Distance (mm)	170	250
Φ (deg.) collected / ΔΦ (deg.)	180/0.25	180/0.2
Exposure time (seconds)	0.25	0.2
Temperature of collect (Kelvin)	100	100
Data statistics		
Space group	$P6_222$	$P2_1$
Unit-Cell parameters (Å)	a=b=133.05 and c=112.45	a = 74.80, b = 104.97 and<br><br>c = 369.61. ß=93.03
Total reflections	122934 (12088)	776402 (68257)
Unique reflections	61467 (6044)	399609 (35839)
Multiplicity	19.4 (18.1)	1.9 (1.9)
Data completeness (%)	100 (99.9)	98.22 (88.1)
I/σ(I)	15.9 (0.7)	4.81 (1.26)
R_{merge} (%)	0.123(1.088)	0.107 (0.518)
CC1/2	0.999 (0.353)	0.982 (0.41)
Structure Refinement		
Reflections used in refinement	54990 (4261)	399362 (35647)

Reflections used for R_{free}	1776 (127)	2001 (171)
R_{factor} (%)	16.6 (37.1)	19.5 (28.6)
R_{free} (%)	18.4 (38.3)	23.4 (32.7)
RMS from ideal geometry		
Bond lengths (Å)	0.004	0.009
Bond angles (º)	0.728	0.635
Average B-factor	40.92	35.02
Macromolecules	40.79	34.86
Solvent	42.41	36.56
Ramachandran Plot		
Favored region (%)	96.2	96.7
Outliers region (%)	0.2	0.2
**PDB ID**	**7T1C**	**7T1J**

Supplementary Data File 1: Spreadsheet containing all DNA sequences and mutagenesis primers used in this study.

REFERENCES AND NOTES

1. T. T. Nguyen, R. Ghirlando, J. Roche, V. Venditti, Structure elucidation of the elusive enzyme I monomer reveals the molecular mechanisms linking oligomerization and enzymatic activity. *Proc. Natl. Acad. Sci. U.S.A.* **118**, e2100298118 (2021).

2. S. H.-C. Yip, J.-L. Foo, G. Schenk, L. R. Gahan, P. D. Carr, D. L. Ollis, Directed evolution combined with rational design increases activity of GpdQ toward a non-physiological substrate and alters the oligomeric structure of the enzyme. *Protein Eng. Des. Sel.* **24**, 861–872 (2011).

3. G. K. A. Hochberg, Y. Liu, E. G. Marklund, B. P. H. Metzger, A. Laganowsky, J. W. Thornton, A hydrophobic ratchet entrenches molecular complexes. *Nature* **588**, 1–6 (2020).

4. M. W. Gray, J. Lukeš, J. M. Archibald, P. J. Keeling, W. F. Doolittle, Irremediable complexity? *Science* **330**, 920–921 (2010).

5. M. Lynch, Evolutionary diversification of the multimeric states of proteins. *Proc. Natl. Acad. Sci. U.S.A.* **110**, E2821–E2828 (2013).

6. S. Dey, D. W. Ritchie, E. D. Levy, PDB-wide identification of biological assemblies from conserved quaternary structure geometry. *Nat. Methods* **15**, 67–72 (2018).

7. E. D. Levy, E. B. Erba, C. V. Robinson, S. A. Teichmann, Assembly reflects evolution of protein complexes. *Nature* **453**, 1262–1265 (2008).

8. E. D. Levy, J. B. Pereira-Leal, C. Chothia, S. A. Teichmann, 3D complex: A structural classification of protein complexes. *PLoS Comput. Biol.* **2**, e155 (2006).

9. E. D. Levy, S. A. Teichmann, Structural, evolutionary, and assembly principles of protein oligomerization, in *Progress in Molecular Biology and Translational Science*, J. Giraldo, F. Ciruela, Eds., vol. 117 of *Oligomerization in Health and Disease* (Academic Press, 2013), pp. 25–51.

10. Y. M. Bar-On, R. Milo, The global mass and average rate of rubisco. *Proc. Natl. Acad. Sci. U.S.A.* **116**, 4738–4743 (2019).

11. G. Schneider, Y. Lindqvist, C. I. Branden, Rubisco: Structure and mechanism. *Annu. Rev. Biophys. Biomol. Struct.* **21**, 119–143 (1992).

12. D. M. Banda, J. H. Pereira, A. K. Liu, D. J. Orr, M. Hammel, C. He, M. A. J. Parry, E. Carmo-Silva, P. D. Adams, J. F. Banfield, P. M. Shih, Novel bacterial clade reveals origin of form I rubisco. *Nat. Plants*, 1–9 (2020).

13. G. Schneider, Y. Lindqvist, T. Lundqvist, Crystallographic refinement and structure of ribulose-1,5-bisphosphate carboxylase from *Rhodospirillum rubrum* at 1.7 Å resolution. *J. Mol. Biol.* **211**, 989–1008 (1990).

14. S. Satagopan, S. Chan, L. J. Perry, F. R. Tabita, Structure-function studies with the unique hexameric form II ribulose-1,5-bisphosphate carboxylase/oxygenase (Rubisco) from *Rhodopseudomonas palustris*. *J. Biol. Chem.* **289**, 21433–21450 (2014).

15. V. A. Varaljay, S. Satagopan, J. A. North, B. Witte, M. N. Dourado, K. Anantharaman, M. A. Arbing, S. H. McCann, R. S. Oremland, J. F. Banfield, K. C. Wrighton, F. R. Tabita, Functional metagenomic selection of ribulose 1, 5-bisphosphate carboxylase/oxygenase from uncultivated bacteria. *Environ. Microbiol.* **18**, 1187–1199 (2016).

16. D. Davidi, M. Shamshoum, Z. Guo, Y. M. Bar-On, N. Prywes, A. Oz, J. Jablonska, A. Flamholz, D. G. Wernick, N. Antonovsky, B. de Pins, L. Shachar, D. Hochhauser, Y. Peleg, S. Albeck, I. Sharon, O. Mueller-Cajar, R. Milo, Highly active rubiscos discovered by systematic interrogation of natural sequence diversity. *EMBO J.*, e104081 (2020).

17. G. L. Hura, A. L. Menon, M. Hammel, R. P. Rambo, F. L. Poole II, S. E. Tsutakawa, F. E. Jenney Jr., S. Classen, K. A. Frankel, R. C. Hopkins, S. Yang, J. W. Scott, B. D. Dillard, M. W. W. Adams, J. A. Tainer, Robust, high-throughput solution structural analyses by small angle x-ray scattering (SAXS). *Nat. Methods* **6**, 606–612 (2009).

18. C. D. Putnam, M. Hammel, G. L. Hura, J. A. Tainer, X-ray solution scattering (SAXS) combined with crystallography and computation: Defining accurate macromolecular structures, conformations and assemblies in solution. *Q. Rev. Biophys.* **40**, 191–285 (2007).

19. S. Classen, G. L. Hura, J. M. Holton, R. P. Rambo, I. Rodic, P. J. McGuire, K. Dyer, M. Hammel, G. Meigs, K. A. Frankel, J. A. Tainer, Implementation and performance of SIBYLS: A dual endstation small-angle x-ray scattering and macromolecular crystallography beamline at the advanced light source. *J. Appl. Cryst.* **46**, 1–13 (2013).

20. J. A. Marsh, S. A. Teichmann, Structure, dynamics, assembly, and evolution of protein complexes. *Annu. Rev. Biochem.* **84**, 551–575 (2015).

21. T. Perica, C. Chothia, S. A. Teichmann, Evolution of oligomeric state through geometric coupling of protein interfaces. *Proc. Natl. Acad. Sci. U.S.A.* **109**, 8127–8132 (2012).

22. M. Kayikci, A. J. Venkatakrishnan, J. Scott-Brown, C. N. J. Ravarani, T. Flock, M. M. Babu, Visualization and analysis of non-covalent contacts using the protein contacts atlas. *Nat. Struct. Mol. Biol.* **25**, 185–194 (2018).

23. J. M. Archibald, J. M. Logsdon, W. F. Doolittle, Recurrent paralogy in the evolution of archaeal chaperonins. *Curr. Biol.* **9**, 1053–1056 (1999).

24. W. F. Doolittle, A ratchet for protein complexity. *Nature* **481**, 270–271 (2012).

25. A. R. Buller, S. Brinkmann-Chen, D. K. Romney, M. Herger, J. Murciano-Calles, F. H. Arnold, Directed evolution of the tryptophan synthase β-subunit for stand-alone function recapitulates allosteric activation. *Proc. Natl. Acad. Sci. U.S.A.* **112**, 14599–14604 (2015).

26. L. Wang, S. Tharp, T. Selzer, S. J. Benkovic, A. Kohen, Effects of a distal mutation on active site chemistry. *Biochemistry* **45**, 1383–1392 (2006).

27. H. Garcia-Seisdedos, C. Empereur-Mot, N. Elad, E. D. Levy, Proteins evolve on the edge of supramolecular self-assembly. *Nature* **548**, 244–247 (2017).

28. R. Zeng, C. Lv, J. Zang, T. Zhang, G. Zhao, Designing stacked assembly of type III rubisco for CO_2 fixation with higher efficiency. *J. Agric. Food Chem.* **70**, 7049–7057 (2022).

29. H. Garcia Seisdedos, T. Levin, G. Shapira, S. Freud, E. D. Levy, Mutant libraries reveal negative design shielding proteins from supramolecular self-assembly and relocalization in cells. *Proc. Natl. Acad. Sci. U.S.A.* **119**, e2101117119 (2022).

30. N. P. King, W. Sheffler, M. R. Sawaya, B. S. Vollmar, J. P. Sumida, I. André, T. Gonen, T. O. Yeates, D. Baker, Computational design of self-assembling protein nanomaterials with atomic level accuracy. *Science* **336**, 1171–1174 (2012).

31. C. Wetzel, S. Pifferi, C. Picci, C. Gök, D. Hoffmann, K. K. Bali, A. Lampe, L. Lapatsina, R. Fleischer, E. S. J. Smith, V. Bégay, M. Moroni, L. Estebanez, J. Kühnemund, J. Walcher, E. Specker, M. Neuenschwander, J. P. von Kries, V. Haucke, R. Kuner, J. F. A. Poulet, J. Schmoranzer, K. Poole, G. R. Lewin, Small-molecule inhibition of STOML3 oligomerization reverses pathological mechanical hypersensitivity. *Nat. Neurosci.* **20**, 209–218 (2017).

32. O. V. Galzitskaya, Oligomers are promising targets for drug development in the treatment of proteinopathies. *Front. Mol. Neurosci.* **12**, 319 (2020).

33. B. Schuster-Böckler, A. Bateman, Protein interactions in human genetic diseases. *Genome Biol.* **9**, R9 (2008).

34. M. L. Choi, S. Gandhi, Crucial role of protein oligomerization in the pathogenesis of Alzheimer's and Parkinson's diseases. *FEBS J.* **285**, 3631–3644 (2018).

35. D. S. Goodsell, A. J. Olson, Structural symmetry and protein function. *Annu. Rev. Biophys. Biomol. Struct.* **29**, 105–153 (2000).

36. K. Hashimoto, A. R. Panchenko, Mechanisms of protein oligomerization, the critical role of insertions and deletions in maintaining different oligomeric states. *Proc. Natl. Acad. Sci. U.S.A.* **107**, 20352–20357 (2010).

37. K. Hashimoto, H. Nishi, S. Bryant, A. R. Panchenko, Caught in self-interaction: Evolutionary and functional mechanisms of protein homooligomerization. *Phys. Biol.* **8**, 035007 (2011).

38. T. J. Andrews, Catalysis by cyanobacterial ribulose-bisphosphate carboxylase large subunits in the complete absence of small subunits. *J. Biol. Chem.* **263**, 12213–12219 (1988).

39. W. Li, A. Godzik, Cd-hit: A fast program for clustering and comparing large sets of protein or nucleotide sequences. *Bioinformatics* **22**, 1658–1659 (2006).

40. K. Katoh, J. Rozewicki, K. D. Yamada, MAFFT online service: Multiple sequence alignment, interactive sequence choice and visualization. *Brief. Bioinform.* **20**, 1160–1166 (2019).

41. D. Darriba, G. L. Taboada, R. Doallo, D. Posada, ProtTest 3: Fast selection of best-fit models of protein evolution. *Bioinforma. Oxf. Engl.* **27**, 1164–1165 (2011).

42. I. Mayrose, D. Graur, N. Ben-Tal, T. Pupko, Comparison of site-specific rate-inference methods for protein sequences: Empirical bayesian methods are superior. *Mol. Biol. Evol.* **21**, 1781–1791 (2004).

43. S. Frey, D. Görlich, A new set of highly efficient, tag-cleaving proteases for purifying recombinant proteins. *J. Chromatogr. A* **1337**, 95–105 (2014).

44. K. N. Dyer, M. Hammel, R. P. Rambo, S. E. Tsutakawa, I. Rodic, S. Classen, J. A. Tainer, G. L. Hura, High-throughput SAXS for the characterization of biomolecules in solution: A practical approach. *Methods Mol. Biol. Clifton NJ.* **1091**, 245–258 (2014).

45. M. D. Tully, N. Tarbouriech, R. P. Rambo, S. Hutin, Analysis of SEC-SAXS data via EFA deconvolution and scatter. *JoVE J. Vis. Exp.*, e61578 (2021).

46. D. Schneidman-Duhovny, M. Hammel, A. Sali, FoXS: A web server for rapid computation and fitting of SAXS profiles. *Nucleic Acids Res.* **38**, W540–W544 (2010).

47. D. Schneidman-Duhovny, M. Hammel, J. A. Tainer, A. Sali, Accurate SAXS profile computation and its assessment by contrast variation experiments. *Biophys. J.* **105**, 962–974 (2013).

48. H. Alonso, M. J. Blayney, J. L. Beck, S. M. Whitney, Substrate-induced assembly of *Methanococcoides burtonii* d-Ribulose-1,5-bisphosphate carboxylase/oxygenase dimers into decamers. *J. Biol. Chem.* **284**, 33876–33882 (2009).

49. J. H. Pereira, R. P. McAndrew, G. P. Tomaleri, P. D. Adams, Berkeley screen: A set of 96 solutions for general macromolecular crystallization. *J. Appl. Cryst.* **50**, 1352–1358 (2017).

50. G. Winter, C. M. C. Lobley, S. M. Prince, Decision making in xia2. *Acta Crystallogr. D Biol. Crystallogr.* **69**, 1260–1273 (2013).

51. A. J. McCoy, R. W. Grosse-Kunstleve, P. D. Adams, M. D. Winn, L. C. Storoni, R. J. Read, Phaser crystallographic software. *J. Appl. Cryst.* **40**, 658–674 (2007).

52. T. C. Terwilliger, R. W. Grosse-Kunstleve, P. V. Afonine, N. W. Moriarty, P. H. Zwart, L.-W. Hung, R. J. Read, P. D. Adams, Iterative model building, structure refinement and density modification with the PHENIX autobuild wizard. *Acta Crystallogr. D Biol. Crystallogr.* **64**, 61–69 (2008).

53. P. D. Adams, P. V. Afonine, G. Bunkóczi, V. B. Chen, I. W. Davis, N. Echols, J. J. Headd, L.-W. Hung, G. J. Kapral, R. W. Grosse-Kunstleve, A. J. McCoy, N. W. Moriarty, R. Oeffner, R. J. Read, D. C. Richardson, J. S. Richardson, T. C. Terwilliger, P. H. Zwart, PHENIX: A comprehensive Python-based system for macromolecular structure solution. *Acta Crystallogr. D Biol. Crystallogr.* **66**, 213–221 (2010).

54. P. V. Afonine, R. W. Grosse-Kunstleve, N. Echols, J. J. Headd, N. W. Moriarty, M. Mustyakimov, T. C. Terwilliger, A. Urzhumtsev, P. H. Zwart, P. D. Adams, Towards automated crystallographic structure refinement with phenix.refine. *Acta Crystallogr. D Biol. Crystallogr.* **68**, 352–367 (2012).

55. P. Emsley, K. Cowtan, Coot: Model-building tools for molecular graphics. *Acta Crystallogr. D Biol. Crystallogr.* **60**, 2126–2132 (2004).

56. I. W. Davis, A. Leaver-Fay, V. B. Chen, J. N. Block, G. J. Kapral, X. Wang, L. W. Murray, W. B. Arendall, J. Snoeyink, J. S. Richardson, D. C. Richardson, MolProbity: All-atom contacts and structure validation for proteins and nucleic acids. *Nucleic Acids Res.* **35**, W375–W383 (2007).

57. A. Prins, D. J. Orr, P. J. Andralojc, M. P. Reynolds, E. Carmo-Silva, M. A. J. Parry, Rubisco catalytic properties of wild and domesticated relatives provide scope for improving wheat photosynthesis. *J. Exp. Bot.* **67**, 1827–1838 (2016).

58. R. E. Sharwood, O. Ghannoum, S. M. Whitney, Prospects for improving CO2 fixation in C3-crops through understanding C4-Rubisco biogenesis and catalytic diversity. *Curr. Opin. Plant Biol.* **31**, 135–142 (2016).

59. A. a. J. Parry, A. J. Keys, S. Gutteridge, Variation in the specificity factor of C3 higher plant rubiscos determined by the total consumption of ribulose-P2. *J. Exp. Bot.* **40**, 317–320 (1989).

60. Y. Song, F. DiMaio, R. Y.-R. Wang, D. Kim, C. Miles, T. Brunette, J. Thompson, D. Baker, High-resolution comparative modeling with RosettaCM. *Structure* **21**, 1735–1742 (2013).

61. F. Madeira, Y. M. Park, J. Lee, N. Buso, T. Gur, N. Madhusoodanan, P. Basutkar, A. R. N. Tivey, S. C. Potter, R. D. Finn, R. Lopez, The EMBL-EBI search and sequence analysis tools APIs in 2019. *Nucleic Acids Res.* **47**, W636–W641 (2019).

62. M. V. Shapovalov, R. L. Dunbrack Jr., A smoothed backbone-dependent rotamer library for proteins derived from adaptive kernel density estimates and regressions. *Structure* **19**, 844–858 (2011).

63. F. Khatib, S. Cooper, M. D. Tyka, K. Xu, I. Makedon, Z. Popovic, D. Baker, F. Players, Algorithm discovery by protein folding game players. *Proc. Natl. Acad. Sci. U.S.A.* **108**, 18949–18953 (2011).

64. S. Chaudhury, S. Lyskov, J. J. Gray, PyRosetta: A script-based interface for implementing molecular modeling algorithms using Rosetta. *Bioinformatics* **26**, 689–691 (2010).

65. R. F. Alford, A. Leaver-Fay, J. R. Jeliazkov, M. J. O'Meara, F. P. DiMaio, H. Park, M. V. Shapovalov, P. D. Renfrew, V. K. Mulligan, K. Kappel, J. W. Labonte, M. S. Pacella, R. Bonneau, P. Bradley, R. L. Dunbrack, R. Das, D. Baker, B. Kuhlman, T. Kortemme, J. J. Gray, The rosetta all-atom energy function for macromolecular modeling and design. *J. Chem. Theory Comput.* **13**, 3031–3048 (2017).

66. X. Robert, P. Gouet, Deciphering key features in protein structures with the new ENDscript server. *Nucleic Acids Res.* **42**, W320–W324 (2014).

67. I. Letunic, P. Bork, Interactive tree of life (iTOL) v5: An online tool for phylogenetic tree display and annotation. *Nucleic Acids Res.* **49**, W293–W296 (2021).

68. T. D. Goddard, C. C. Huang, E. C. Meng, E. F. Pettersen, G. S. Couch, J. H. Morris, T. E. Ferrin, UCSF ChimeraX: Meeting modern challenges in visualization and analysis. *Protein Sci. Publ. Protein Soc.* **27**, 14–25 (2018).

69. E. F. Pettersen, T. D. Goddard, C. C. Huang, E. C. Meng, G. S. Couch, T. I. Croll, J. H. Morris, T. E. Ferrin, UCSF ChimeraX: Structure visualization for researchers, educators, and developers. *Protein Sci.* **30**, 70–82 (2021).

70. S. Knight, I. Andersson, C.-I. Brändén, Crystallographic analysis of ribulose 1,5-bisphosphate carboxylase from spinach at 2·4 Å resolution: Subunit interactions and active site. *J. Mol. Biol.* **215**, 113–160 (1990).
